# Transcriptome of neonatal preBötzinger complex neurones in Dbx1 reporter mice

**DOI:** 10.1038/s41598-017-09418-4

**Published:** 2017-08-17

**Authors:** John A. Hayes, Andrew Kottick, Maria Cristina D. Picardo, Andrew D. Halleran, Ronald D. Smith, Gregory D. Smith, Margaret S. Saha, Christopher A. Del Negro

**Affiliations:** 1 Department of Applied Science, Integrated Science Center, 540 Landrum Dr., The College of William and Mary, Williamsburg, VA 23185 USA; 2 Department of Biology, Integrated Science Center, 540 Landrum Dr., The College of William and Mary, Williamsburg, VA 23185 USA

## Abstract

We sequenced the transcriptome of brainstem interneurons in the specialized respiratory rhythmogenic site dubbed preBötzinger Complex (preBötC) from newborn mice. To distinguish molecular characteristics of the core oscillator we compared preBötC neurons derived from Dbx1-expressing progenitors that are respiratory rhythmogenic to neighbouring non-Dbx1-derived neurons, which support other respiratory and non-respiratory functions. Results in three categories are particularly salient. First, Dbx1 preBötC neurons express κ-opioid receptors in addition to μ-opioid receptors that heretofore have been associated with opiate respiratory depression, which may have clinical applications. Second, Dbx1 preBötC neurons express the hypoxia-inducible transcription factor *Hif1a* at levels three-times higher than non-Dbx1 neurons, which links core rhythmogenic microcircuits to O_2_-related chemosensation for the first time. Third, we detected a suite of transcription factors including *Hoxa4* whose expression pattern may define the rostral preBötC border, *Pbx3* that may influence ipsilateral connectivity, and *Pax8* that may pertain to a ventrally-derived subset of Dbx1 preBötC neurons. These data establish the transcriptomic signature of the core respiratory oscillator at a perinatal stage of development.

## Introduction

Neural rhythms that drive inspiratory breathing movements in mammals originate from the brainstem preBötzinger complex (preBötC)^1, 2^. Neurons derived from Dbx1-expressing progenitors comprise its rhythmogenic core^3–9^. Although we know the site and neuronal constituents at the point of origin of respiratory rhythm, the cellular and molecular mechanisms that generate and control respiration remain incompletely understood.

Electrophysiological recordings in preBötC neurons generally, and Dbx1-derived preBötC neurons in particular, have characterized intrinsic membrane properties, including ion channels, membrane pumps and transporters, as well as synaptic currents that influence the neural mechanisms of respiration^2, 10, 11^. However, testing their relative rhythm- and pattern-generating roles typically relies on promiscuous pharmacology and leads to inconclusive results. We argue that identifying specific subunits, isoforms, and genes that underlie putatively rhythmogenic conductances and integral membrane proteins would facilitate more conclusive experiments. Knowledge of the newborn mouse preBötC transcriptome – the expressed transcripts and their relative quantity – could be exploited to develop targeted physiological experiments, with the added benefit of uncovering novel genes that may influence preBötC development as well as regulate respiratory function.

Here we provide the first RNA-Seq gene expression profile for preBötC neurons in newborn mice. We analysed gene expression levels within the Dbx1 population as well as differential expression between Dbx1 and non-Dbx1-expressing populations, and we interpret their significance for defining the structure and function of the preBötC in the context of existing literature. These data are publicly available in an open access database (NCBI gene expression omnibus, https://www.ncbi.nlm.nih.gov/geo/) for custom analyses and applications that interrogate preBötC development as well as the cellular and molecular neural bases for breathing behaviour.

## Results and Discussion

**Figure 1:**
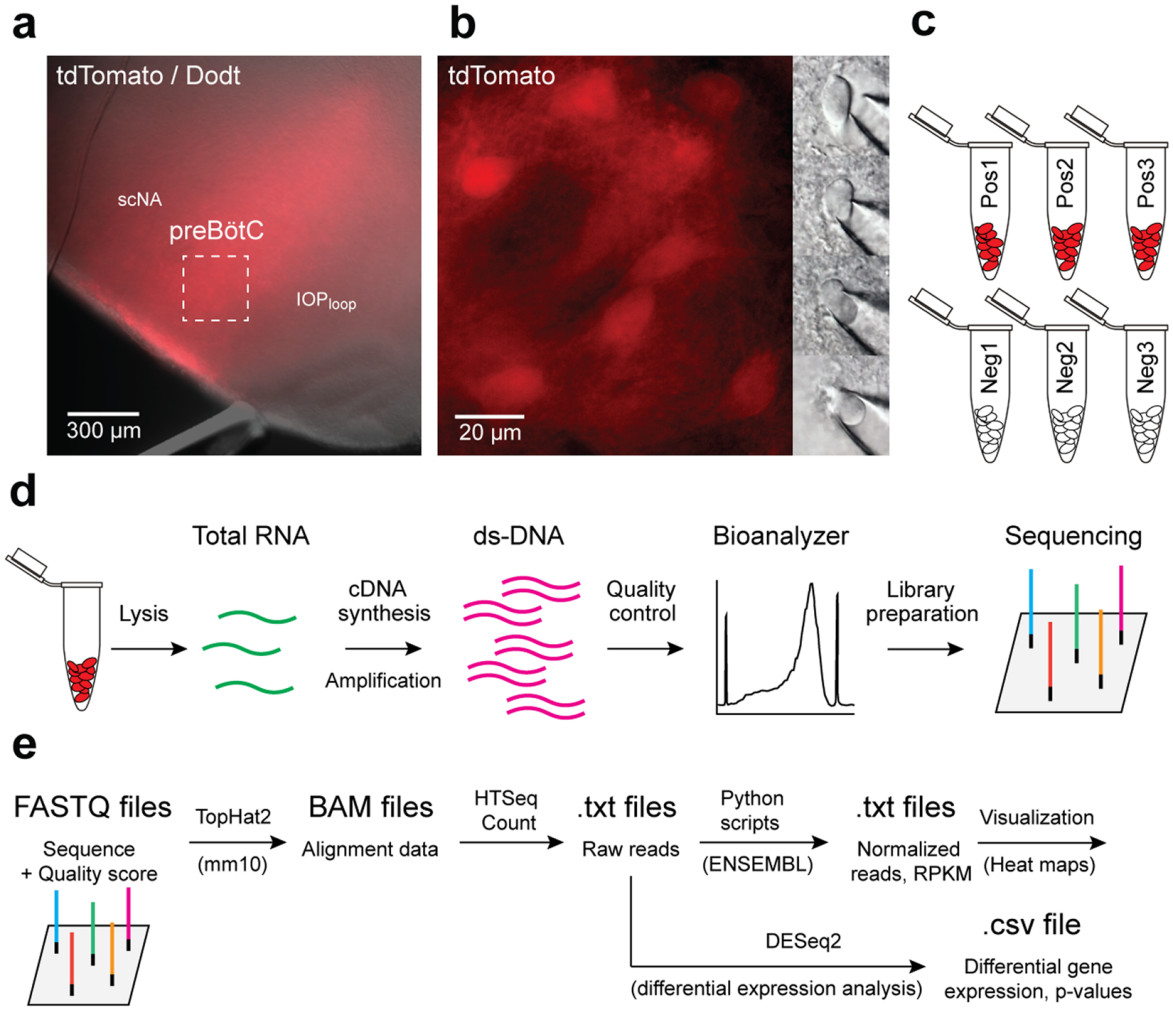
Schematic explanation of experiments and analyses. (**a**) Transverse medullary slice from a neonatal mouse containing the preBötC with tdTomato labelling in Dbx1-derived neurons. (**b**) Magnification from panel **a** showing tdTomato expressing Dbx1 neurons and an extraction on the right performed under Dodt imaging. (**c**) Six samples (three Dbx1, three non-Dbx1) were acquired. Each sample consists of 15 neurons. (**d**) RNA-Seq workflow as detailed in the text. (**e**) Bioinformatic workflow as detailed in the text.

We identified Dbx1-derived neurons (hereafter, Dbx1 neurons) in neonatal mouse preBötC slices by tdTomato fluorescence, which resulted from crossing the Dbx1 Cre-driver strain, *Dbx1*^CreERT2^ with a floxed responder strain, *Rosa26*^tdTomato^, and then preparing transverse slices that expose the preBötC. We harvested 15 preBötC neurons per sample. We collected three separate Dbx1 samples (Pos1, Pos2, and Pos3) and three separate non-Dbx1 neuron samples (Neg1, Neg2, and Neg3), *i.e*., six samples from six different animals in total (Fig. 1a–c).

**Figure 2:**
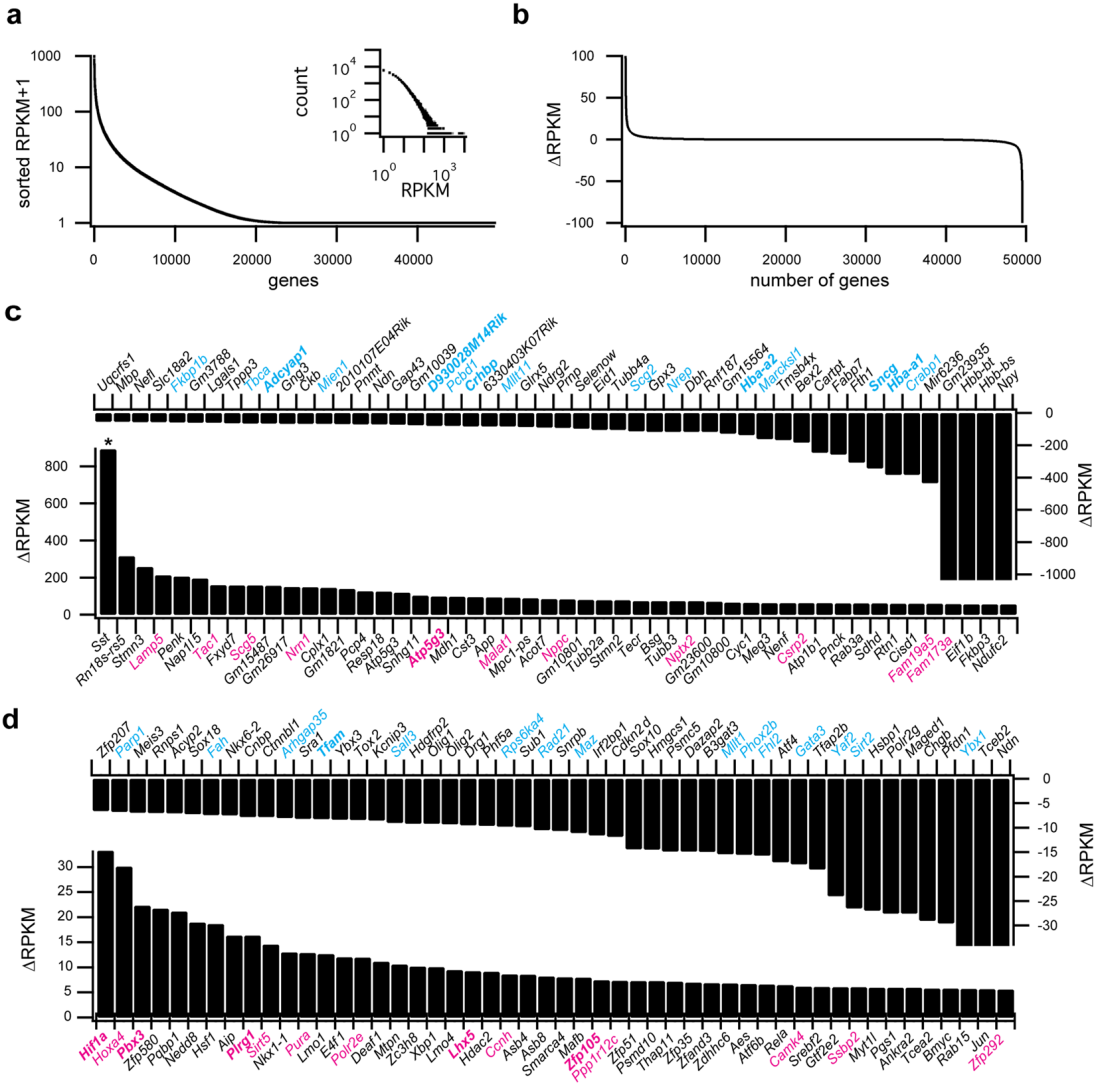
Summary of RNA-Seq data. (**a**) RPKMs sorted by numerical mean value for each gene across the 49,568 genes in the *Ensembl* database. Inset shows the distribution of all RPKM values (148,704 values for each the Dbx1 and non-Dbx1 sample sets) in 1000 bins, which conforms to a power law. Count on the ordinate refers to the number of genes in each bin. (**b**) Difference in RPKMs between Dbx1 and non-Dbx1 samples for all genes (*i.e*., ∆RPMK = RPKM_Dbx1_ − RPKM_non-Dbx1_). (**c**) Top portion shows a detail from panel b featuring the 50 genes with greatest expression in non-Dbx1 samples (∆RPMK < 0). Bottom portion shows a detail of the 50 genes with greatest expression in Dbx1 samples (∆RPMK > 0). (**d**) Difference in RPKMs between Dbx1 and non-Dbx1 samples for 50 transcription factors with greatest expression in non-Dbx1 samples (top, ∆RPMK < 0) and Dbx1 samples (bottom, ∆RPMK > 0). For both **c** and **d**, if L2FC > 0, then genes for which p < 0.05 are labelled in plain magenta typeface and those at FDR < 0.1 are labelled in bold magenta typeface; if L2FC < 0, then genes at p < 0.05 are labelled in plain cyan typeface and those at FDR < 0.1 are labelled in bold cyan typeface.

We employed RNA-Seq (Fig. 1d,e) to identify 49,568 gene candidates in the murine *Ensembl* database^12^ expressed in both Dbx1-derived and non-Dbx1-derived preBötC neurons, including 22,050 protein-coding genes. All of the genes belong to one of 43 biotypes, which includes pseudogenes, long non-coding^13^ and short non-coding RNAs^14^, as well as predicted genes^12^ (Table 1). The distribution of reads per kilobase of transcript per million mapped reads (RPKM) follows a power law (Fig. 2a and its inset) as expected for RNA-Seq and microarray data^15^. The median and mean for RPKM are 1.74 and 11.43, respectively.

**Table 1:** The number of genes with non-zero reads for Dbx1 (Pos1, Pos2, Pos3) and non-Dbx1 (Neg1, Neg2, Neg3) samples by biotype. ‘Total’ refers to the total number of genes in the *Ensembl* database of each biotype.

biotype	Total	Pos1	Pos2	Pos3	Neg1	Neg2	Neg3
**Protein Coding**							
IG_C_gene	13	2	1	1	1	1	0
IG_D_gene	19	0	0	0	0	0	0
IG_J_gene	14	0	0	0	0	0	0
IG_LV_gene	2	0	0	0	0	0	0
IG_V_gene	218	4	9	0	6	3	3
TEC	2690	607	941	710	708	647	473
TR_C_gene	8	0	0	1	1	0	1
TR_D_gene	4	0	0	0	0	0	0
TR_J_gene	70	0	6	0	1	3	1
TR_V_gene	144	5	4	0	5	3	2
polymorphic_pseudogene	69	6	6	4	11	10	6
protein_coding	22021	13026	13650	12849	13409	13457	12541
**Pseudogenes**							
IG_C_pseudogene	1	0	0	0	0	0	0
IG_D_pseudogene	3	0	0	0	0	0	0
IG_V_pseudogene	155	2	0	1	3	3	1
IG_pseudogene	2	0	0	0	0	0	0
TR_J_pseudogene	10	0	1	0	0	0	0
TR_V_pseudogene	34	0	1	0	0	0	0
processed_pseudogene	7282	2058	2111	1878	1893	2100	2013
pseudogene	100	32	36	25	26	28	28
transcribed_processed_pseudogene	197	72	86	82	79	79	67
transcribed_unitary_pseudogene	8	2	6	3	3	3	3
transcribed_unprocessed_pseudogene	191	38	36	41	35	36	30
unitary_pseudogene	26	7	8	10	9	6	6
unprocessed_pseudogene	2433	92	107	89	86	86	82
**Long non-coding RNAs**							
3prime_overlapping_ncRNA	2	0	0	0	0	0	0
antisense	2415	443	634	517	492	519	378
bidirectional_promoter_lncRNA	61	21	27	19	18	25	19
lincRNA	4247	532	728	537	630	598	438
macro_lncRNA	1	0	0	0	0	0	0
processed_transcript	749	240	295	243	236	238	199
sense_intronic	270	62	89	81	68	67	47
sense_overlapping	25	6	7	7	7	9	10
**Short non-coding RNAs**							
Mt_rRNA	2	0	0	0	0	0	0
Mt_tRNA	22	0	0	0	0	0	0
miRNA	2201	56	106	83	82	84	63
misc_RNA	564	34	33	27	27	23	20
rRNA	354	9	10	9	9	12	9
ribozyme	22	4	6	3	4	3	3
sRNA	2	0	0	0	0	0	0
scRNA	1	0	0	0	0	0	0
scaRNA	46	6	9	9	5	7	6

There were 23,263 genes with aligned reads (*i.e*., non-zero RPKM) in the Dbx1 samples and 23,015 genes in the non-Dbx1 samples. Figure 2b plots ∆RPKM (RPKM_*Dbx1*_ – RPKM_*non-Dbx1*_). The left knee illustrates genes more highly expressed in the Dbx1 samples, whereas the right knee illustrates genes more highly expressed in the non-Dbx1 samples (the knees of Fig. 2b are depicted at higher resolution in Fig. 2c).

**Figure 3:**
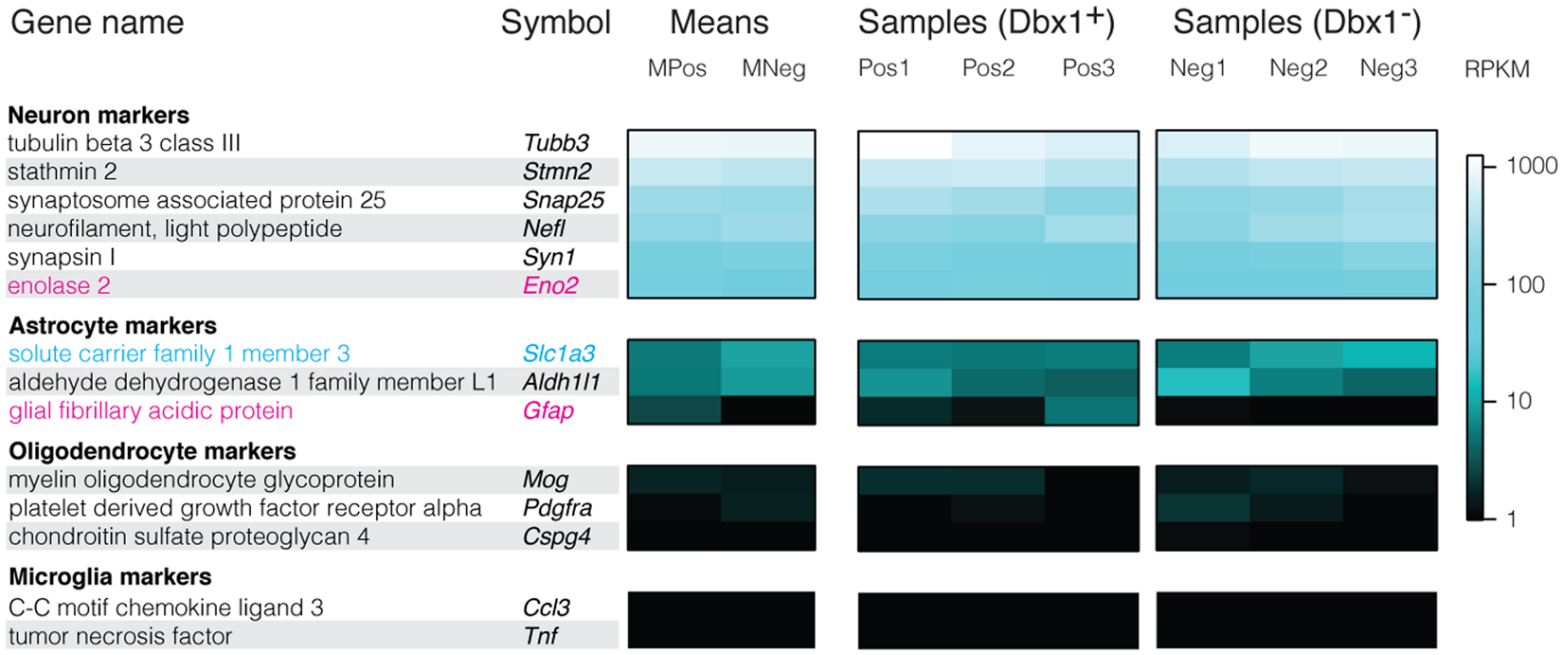
Heat maps showing relative levels of neuronal and glial marker expression. Gene names and symbols are organized according to cell type. Mean RPKM of samples, and RPKM of each sample is indicated by a pseudo-colour scale (right). Genes for which L2FC > 0 and p < 0.05 are labelled in magenta typeface. Genes for which L2FC < 0 and p < 0.05 are labelled in cyan typeface.

Dbx1-expressing progenitors give rise to preBötC neurons and glia^16^. Both Dbx1 and non-Dbx1 samples expressed neuronal marker genes such as synapsin 1, Snap25, and Tubb3 (among others) at levels exceeding the median RPKM by more than two orders of magnitude (we combined Dbx1 and non-Dbx1 samples to assess neuronal markers in general; RPKM measured 313.1 ± 299.2, mean ± SD). Non-neuronal cell marker genes were undetectable or minimally expressed (we similarly combined Dbx1 and non-Dbx1 samples to assess glial markers; RPKM measured 1.7 ± 3.2, mean ± SD)^17, 18^. Thus, our Dbx1 and non-Dbx1 samples reflect neurons as opposed to glia (Fig. 3).

### Amino acid neurotransmitters

Excitatory preBötC neurons are respiratory rhythmogenic^19–25^. Inhibitory neurons, which populate the preBötC in roughly equal numbers, regulate respiratory rhythm and mediate sensorimotor integration^26–29^ (*q.v*., refs 11, 30).

Given the well-established role of Dbx1 preBötC neurons in rhythm generation^3, 4, 6–9^, we expected the Dbx1 samples to express transcripts associated with excitatory transmitter phenotype. By contrast, we expected non-Dbx1 samples to exhibit markers of both excitatory and inhibitory transmitter phenotypes.

The log_2_ fold change (L2FC) quantifies the relative level of expression between Dbx1 and non-Dbx1 neurons. L2FC > 0 for genes more highly expressed in Dbx1 neurons; L2FC < 0 for genes more highly expressed in non-Dbx1 neurons. However, there is no statistical test associated with L2FC *per se*. We evaluated differential expression between the two populations via a Wald test that computes a raw probability and a false discovery rate (FDR) that adjusts for multiple comparisons (see Methods).

Dbx1 neurons expressed glutamate-synthesizing enzymes, transporters, and receptors (Supplementary Fig. S1a). *Slc17a6*, encoding Vglut2, was expressed more than two-fold higher in Dbx1 compared to non-Dbx1 neurons; the associated L2FC measured 1.28. Of all 49,568 genes examined, the raw RPKM difference (*i.e*., ∆RPKM) for *Slc17a6* rank 61^st^. Nevertheless, *Slc17a6* was not differentially expressed according to the orthodox threshold of FDR < 0.1 (p = 0.0066, FDR = 0.29). The Benjamini-Hochberg correction^31^ used to calculate FDR aggressively combats Type I errors, *i.e*., false positive discoveries^31, 32^, at the expense of inflating false negatives (Type II errors)^33^. We argue that the FDR in the case of *Slc17a6* most likely reflects a Type II error because an array of independent studies demonstrate that Dbx1 neurons are the predominant source of glutamatergic neurons within the preBötC^3, 4, 7, 8^.

Contrary to our expectations, gene expression for inhibitory amino acid-synthesizing enzymes and transporters was commensurate for Dbx1 and non-Dbx1 neurons (Supplementary Fig. S1b), quantified by L2FC ~ 0. For *Slc6a5*, encoding glycine transporter 2, *i.e*., GlyT2, L2FC measured −0.11 (p = 0.84, FDR = 1.0). For glutamic acid decarboxylase 1, *i.e*., *Gad1*, encoding GAD1, L2FC_*Gad1*_ measured −0.28 (p = 0.62, FDR = 1.0). Finally, for glutamic acid decarboxylase 2, *i.e*., *Gad2*, encoding GAD2, L2FC_*Gad2*_ measured −0.34 (p = 0.35, FDR = 1.0).

The RNA related to inhibitory synaptic transmission may remain untranslated in most Dbx1 preBötC neurons similar to excitatory CA1 hippocampal neurons where post-transcriptional regulation controls neurotransmitter phenotype^34^. Nevertheless, a non-negligible subset of Dbx1 preBötC neurons communicate via chloride-mediated synaptic inhibition. For example, Gray *et al*. (ref. 4) identified inhibitory Dbx1 preBötC neurons that expressed GAD1 or GlyT2. Dbx1 preBötC neurons with inhibitory transmitter phenotype would be well equipped to periodically suppress activity in respiratory nuclei such as the expiratory-related lateral parafacial respiratory group^35–38^ or the post-inspiratory complex^39^, both of which discharge neural bursts out-of-sync with the preBötC. It is also possible that inhibitory Dbx1 preBötC neurons suppress orofacial behaviours during the inspiratory phase of the breathing cycle^40, 41^ or participate in inhibitory pulmonary stretch receptor feedback.

### Peptides and peptide receptors

Peptide and peptide receptor expression differentiates the preBötC from neighbouring cell groups^42–49^. *Tacr1* and *Tacr3*, which encode tachykinin (*i.e*., neurokinin) receptors were expressed in both Dbx1 and non-Dbx1 preBötC neurons but there was no evidence of differential expression (L2FC_*Tacr1*_ = 1.09, p = 0.08, FDR = 0.83; L2FC_*Tacr3*_ = 0.50, p = 0.42, FDR = 1.0, Supplementary Fig. S2). These data are consistent with neurokinin receptor expression being a useful, but not exclusive^45^, marker of rhythmogenic preBötC neurons^44, 48, 50, 51^.

The endogenous ligands for neurokinin 1 receptors, neurokinin A and substance P, both result from tachykinin precursor 1 (*Tac1*), which is highly expressed in both sample populations (RPKM > 60, Supplementary Fig. S2) but *Tac1* did not meet the criterion for differential expression (L2FC = 1.36, p = 0.018, FDR = 0.48). This result is not surprising given that substance P is a cotransmitter in overlapping populations that modulate preBötC function^46, 52, 53^.

Opiate-induced respiratory depression is mediated, in part, by pre- and post-synaptic effects of μ-opioid-receptor activation in the preBötC^44, 54–58^. Both Dbx1 and non-Dbx1 preBötC neurons expressed μ-opioid receptor transcript *Oprm1*, but at levels less than the median RPKM across all expressed genes (0.74 for *Oprm1* vs. the population median of 1.74). *Oprm1* was not differentially expressed (L2FC = 0.55, p = 0.29, FDR = 1.0). It is unclear whether other opioid receptors contribute to respiratory depression, but it remains a realistic possibility because the μ-opioid agonist DAMGO ([D-Ala^2^, *N*-MePhe^4^, Gly-ol]-enkephalin), which has potent effects *in vitro*^44^, also acts at δ- and κ-opioid receptors^59^. Fentanyl, used *in vivo* because it crosses the blood-brain barrier, activates μ- and κ-opioid receptors^60^. The κ-opioid receptor transcript *Oprk1* was expressed in both Dbx1 and non-Dbx1 neurons. Although *Oprk1* expression level appeared much higher than *Oprm1* (RPKM_*Oprk1*_ = 2.61 vs. RPKM_*Oprm1*_ = 0.74) this difference was not statistically significant by the Wilcoxon-Mann-Whitney U-test (p = 0.09, Supplementary Fig. S2). *Oprk1* was commensurately expressed in Dbx1 and non-Dbx1 preBötC neurons (L2FC = 0.92, p = 0.16, FDR = 0.97). κ-opioid receptors have been associated with stress and anxiety^61^, but their expression in preBötC neurons suggests they may be relevant to opiate neuromodulation of respiratory rhythm or behaviours linked to respiration.

Somatostatin (*Sst*) and somatostatin receptor (*Sstr1*, *Sstr2, Sstr3*, *Sstr5*) expression characterize preBötC neurons that serve obligatory rhythmogenic or premotor functions^7, 8, 44, 47–49, 62–64^. Of all our transcriptomic data from both Dbx1 and non-Dbx1 preBötC neurons, *Sst* had the highest expression level of any gene (*in Supplementary Fig. S2 and Fig. 2c), but it was not differentially expressed (L2FC = 0.92, p = 0.14, FDR = 0.93). Somatostatin receptor genes *Sstr1*, *Sstr2*, *Sstr3*, and *Sstr5* were expressed too at lower levels (Supplementary Fig. S2).

### Transcription factors and other distinguishing markers of preBötC Dbx1 and non-Dbx1 neurons

**Figure 4:**
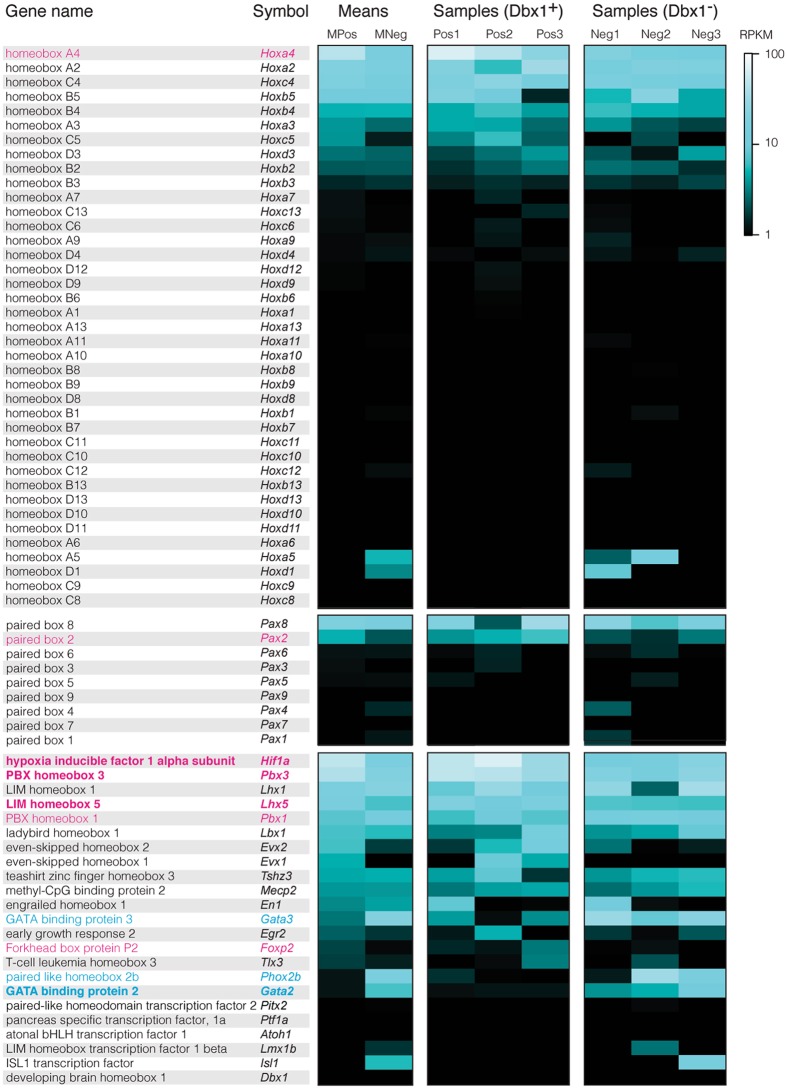
Heat maps showing relative levels of selected transcription factors in three categories: *Hox* genes, *Pax* genes, and other genes. Transcription factors are sorted in descending order according to mean RPKM in the Dbx1 samples. If L2FC > 0, then genes at p < 0.05 for differential expression are labelled in plain magenta typeface and those at FDR < 0.1 are labelled in bold magenta typeface. If L2FC < 0, then genes at p < 0.05 are labelled in plain cyan typeface and those at FDR < 0.1 are labelled in bold cyan typeface. RPKM is indicated by a pseudo-colour scale (right).

Combinatorial codes of transcription factors, expressed in the developing spinal cord and hindbrain, govern the assembly of central pattern generating circuits for locomotion and breathing^65–68^. We cross-referenced our RNA-Seq results with a list of murine transcription factors (RIKEN Institute, Wako, Japan) as well as the curated TF-checkpoint project^69^ to determine the transcription factors expressed in Dbx1 and non-Dbx1 neurons. We detected 1,281 transcription factors among protein-coding genes. Figure 2d depicts 100 transcription factors sorted according to the difference in their expression levels (∆RPKM). Figure 4 shows heat maps for transcription factor expression in each Dbx1 and non-Dbx1 neuron sample. Transcription factors differentially expressed in the Dbx1 population include *Hif1a* (L2FC = 1.52, p = 0.0001, FDR = 0.022) and *Pbx3* (L2FC = 1.06, p = 0.00093, FDR = 0.093). *Hif1a* and *Pbx3* occupy the first and third positions of Fig. 2d (lower plot).

HIF-1 (hypoxia-inducible factor 1, a protein complex) regulates gene transcription cascades in response to hypoxia^70–72^. Its expression increases within minutes under hypoxic conditions^73^. We constantly perfused oxygenated solution through the tissue as we isolated neurons from the superficial 40 µm of the tissue slices (e.g., Fig. 1b) wherein there is no diminution of the oxygen gradient^74, 75^. *Hif1a*, differentially-expressed at higher levels in Dbx1 neurons, encodes the O_2_-sensing component of the complex (the HIF-1α subunit). In heterozygotic *Hif1a* knockouts, the carotid body, the primary sensor of arterial O_2_, no longer reacts to hypoxia, which is lethal^76^. To our knowledge HIF-1α has not been associated with respiratory rhythm generation directly, although its upregulation in brainstem may occur at high-altitudes during hypoxic conditions^77^. HIF-1α being highly- and differentially expressed in core rhythmogenic neurons suggests that Dbx1 preBötC neurons may be programmed to increase ventilation in response to hypoxia.

HIF-1α is widely associated with angiogenesis^78^. preBötC neurons are embedded within a network of arterioles^79^ like chemosensitive retrotrapezoid neurons^80^, which suggests that preBötC neurons, like their retrotrapezoid counterparts, need access to blood for chemosensation.

**Figure 5:**
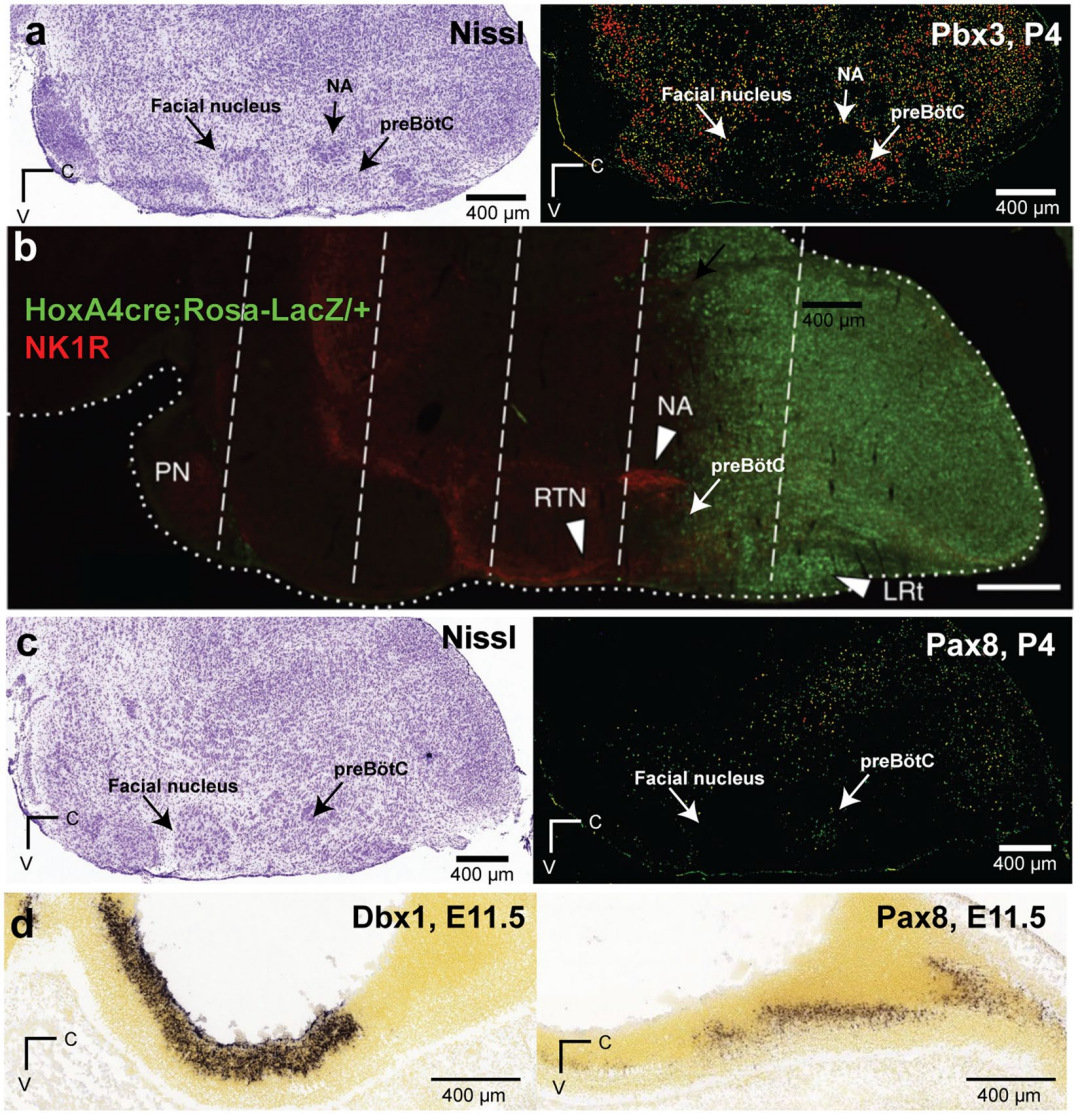
Transcription factors in the hindbrain and preBötC. (**a**) Nissl-stained section (left) and corresponding section labelled by *in situ* hybridization for *Pbx3* (Allen Institute Developing Mouse Brain Atlas [AIDMBA], *Pbx3* at P4, slide 10, expression *in situ* is codified by green-red pseudo-colour scale by AIDMBA). (**b**) *Hoxa4*-expressing brainstem cells (green) in a parasagittal section with NK1R (*Tacr1*) labelling in red. Modified from Fig. 2 of Huang *et al*., 2012 (ref. 92), used with permission. (**c**) Nissl-stained section (left) and corresponding section labelled by *in situ* hybridization for *Pax8* (AIDMBA, *Pax8* at P4, slide 11). (**d**) Parasagittal sections of the mouse brainstem at E11.5 showing *Dbx1* and *Pax8* expression, respectively (AIDMBA, *Dbx1* at E11.5, slide 9; *Pax8* at E11.5, slide 9).

*Pbx3* is a *Hox* gene cofactor^81^. The expression pattern for *Pbx3* is mosaic in transverse sections of the newborn mouse medulla at the level of the preBötC^82^ as well as in parasagittal sections from the Allen Institute developing mouse brain atlas (AIDMBA) (Fig. 5a, AIDMBA: P4, *Pbx3*, slide 10). *Dbx1* and *Pbx3* knockout-mice both die perinatally due to central hypoventilation^3, 4, 82^. *Dbx1* knockout mice never breathe and form no preBötC^3, 4^. In contrast, the *Pbx3* knockout mice breathe irregularly with periodic apnoea, which indicates that the preBötC is present but dysfunctional. *Pbx3* was shown to interact with other *Pbx* genes and *Hox* genes to influence connectivity of motor neurones in ipsilateral motor pools^83^. We posit that *Pbx3* may influence ipsilateral connectivity in the preBötC. Commissural connectivity, in contrast, is governed by *Robo3* in Dbx1 preBötC neurons^3^, which was ostensibly undetected (Dbx1 samples, RPKM = 0.06 ± 0.06; non-Dbx1 samples, RPKM = 0.01 ± 0.02) most likely because commissural projections are mature by early post-natal development. Failure to properly interconnect locally could impair emergent network rhythmicity^6, 24^, and impede normal respiration in *Pbx3*-deficient mice.

*Mafb* (in the 27^th^ position of Fig. 2d, lower) is essential for neonatal breathing; the preBötC in mice lacking *Mafb* suffers severe anatomical deficits, including overall cell loss^84^. No evidence indicates differential expression of *Mafb* in the Dbx1 preBötC population (L2FC = 0.25, p = 0.57, FDR = 1.0).

*Phox2b* is a key regulator of autonomic visceral reflex pathways^85–87^ as well as cardiorespiratory-related chemosensitive circuitry^88–90^. *Phox2b* was detected in 2 of 3 non-Dbx1 samples, undetected in 2 of 3 Dbx1 samples, and minimally detected in the third Dbx1 sample. However, *Phox2b* did not meet the criterion for differential expression (L2FC = −1.75, p = 0.0060, FDR = 0.28, Fig. 2d [36^th^ position, upper plot] and Fig. 4).

Homeobox (Hox) genes govern cell fate identity along the anterior-posterior axis^91^. We observed a cascade of expression among the *Hox2*–*5* genes across the *Hoxa*, *Hoxb*, *Hoxc*, and *Hoxd* chromosomal clusters, which peaked with *Hoxa4* (Fig. 4). *Hoxa4* showed the second highest ∆RPKM among all transcription factors (Fig. 2d, lower plot, and Fig. 4), but it did not pass the threshold for differential expression (L2FC = 1.39, p = 0.0022, FDR = 0.16). In the anterior-posterior axis of the lower medulla, *Hoxa4* expression stops at the caudal border of the compact division of the nucleus ambiguus, which coincides with the rostral limit of the preBötC (Fig. 5b, reproduced with permission from ref. 92), and thus *Hoxa4* expression might influence the rostral preBötC boundary, which would apply to both Dbx1 and non-Dbx1 neurons. *Hox* genes that are also relevant to respiration, in the cervical spinal cord and not the brainstem, include *Hoxa5* and *Hoxc5*^93^, which influence phrenic motoneuron development and survival. That these genes continue to be expressed postnatally to maintain the organization of phrenic motor columns may be generalizable to postnatal expression of *Hox* genes in the preBötC postnatally, but that remains to be investigated.

Paired box (*Pax*) genes influence cell lineage specification^94^. *Pax8* was the highest expressed transcription factor of its class (Fig. 4) in both Dbx1 and non-Dbx1 neurons (L2FC = 0.17, p = 0.79, FDR = 1.0), which is notable because *Pax8* expression perdures in ventral hindbrain neurons in adult mice^95^. Data from the AIDMBA show *Pax8* expression clustered in the vicinity of the preBötC at P4 (Fig. 5c; *Pax8*, slide 11) and *Pax8* expression is on the ventral edge of Dbx1 expression at E11.5 (Fig. 5d and e, AIDMBA: *Dbx1*, slide *9*, and *Pax8*, slide 9). *Pax7* was completely undetected (Fig. 4). *Pax2* had the highest L2FC among *Pax* genes but did not meet the criterion for differential expression (L2FC = 1.39, p = 0.0067, FDR = 0.30). Even though *Pax8* and *Pax2* were not differentially expressed, these data are consistent with core preBötC rhythmogenic neurons making up the ventral subset of V0 interneurons (*i.e*., V0_V_), which express Dbx1 as well as *Pax8* and *Pax2*, but not *Pax7*. In contrast, Dbx1-derived dorsal interneurons (V0_D_), which may reside in the reticular formation and not the preBötC, express *Pax7*^3, 96–98^.

*Dbx1* was undetected in preBötC neurons (Fig. 4) because its expression is restricted to embryonic development (E9.5-E11.5)^3, 4, 16, 96^. Two of the Dbx1 samples (Pos2 and Pos3) exhibited *Evx1* expression for a mean RPKM of 5.76. The third Dbx1 sample (Pos1) did not show *Evx1* expression. *Evx1* was not detected in any non-Dbx1 samples. Because four of the six samples did contain detectable *Evx1*, DESeq. 2 cannot compute statistics beyond raw reads. Nevertheless, we suspect that a significant subset of Dbx1-expressing progenitor cells differentiate into *Evx1*-expressing ventral (V0_V_) interneurons, which ultimately form the preBötC core. In the lumbar spinal cord, *Evx1* expression is critical for V0_V_ interneuron identity^99^. Commissural V0_V_ interneurons in zebrafish spinal cord require *Evx1* (and *Evx2*) to become glutamatergic^100^. We posit that *Evx1* may play analogous roles in Dbx1-derived interneurons of the preBötC.

One Dbx1 sample (Pos1) expressed *En1*, which defines canonical V1 interneurons^101–103^. *En1* expression in the ventral medulla at early postnatal stages overlaps with the preBötC so one of our samples may have inadvertently included *En1*-expressing interneurons (AIDMBA, P1, engrailed 1, *i.e*., *En1*, slides 52–54), which would also explain the lack of *Evx1* expression in Pos1, since these two transcription factors (Evx1 and En1) are mutually exclusive in V0 and V1 interneurons, respectively^102^.

Within the non-Dbx1 population, we found two differentially expressed genes associated with inhibitory neurons. *Gata2* (L2FC = −3.78, p = 2.57E-12, FDR = 7.62E-09,) specifies inhibitory V2b interneurons in spinal cord locomotor circuits^104^. Also, the 5-HT_1A_ receptor (*Htr1a*, L2FC = −2.37, p = 0.000031, FDR = 0.009) is associated with glycinergic (*i.e*., non-Dbx1) preBötC neurons^105^. These data suggest that non-Dbx1 preBötC neurons are inhibitory, and thus could transiently suppress activity expiratory parafacial neurons^35–38^ or the post-inspiratory interneurons^39^, which are silent during preBötC inspiratory bursts. Inhibitory non-Dbx1 preBötC neurons might also coordinate inspiratory rhythm with whisking and other orofacial behaviors^40, 41^.

Neither Dbx1 nor non-Dbx1 preBötC neurons express *Atoh1* (Fig. 4), which is an embryonic transcription factor associated with progenitors of the central chemoreceptive ventral parafacial (pF_V_) or retrotrapezoid nucleus^43, 106–108^. We observed low expression of *Tlx3* and *Egr2* (a.k.a., *Krox20* [ref. 109]). The combined RPKM of *Tlx3* and *Egr2* measured 0.78 ± 1.20. Those data are not surprising because *Tlx3* and *Egr2* are associated with the parafacial and serotonergic raphé neurons, but not preBötC neurons. *Tshz3* and *Mecp2*, which are linked to respiratory-related dysfunction, and the latter specifically with Rett syndrome^110–112^, showed modest expression across both Dbx1 and non-Dbx1 neurons. Their combined RPKM measured 3.33 ± 1.62.

A notable gene, which is not a transcription factor, but was significantly differentially expressed in Dbx1 samples was synaptotagmin-10 (*Syt10*, L2FC_*Syt10*_ = 2.34, p = 0.00034, FDR = 0.047), which is involved in synaptic exocytosis^113, 114^, and could be involved in synaptic depression observed in preBötC rhythmogenic neurons^24, 115^.

### Neonatal preBötC transcriptome provides a baseline for comparative analyses during development

We present this transcriptome, including non-coding transcripts, from perinatal Dbx1 preBötC neurons, which are obligatory rhythm- and pattern-generating interneurons for breathing. We also present the transcriptome of non-Dbx1 preBötC neurons, which serve non-rhythmogenic respiratory and non-respiratory functions. We analysed the perinatal transcriptome because animals at this age are widely employed in respiratory neurobiology research, and provide testable predictions for experiments in adult rodents^2, 11, 116, 117^. Further, brainstem tissue explants from neonatal fluorescent reporter mice are optimal for visual identification and isolation of single cells. These data can be exploited or meta-analysed to design new experiments and approaches that manipulate Dbx1 and non-Dbx1 neuron development and physiology and thus elucidate their roles in the neural generation and control of breathing.

## Methods

### Animals

All procedures were approved by the Institutional Animal Care and Use Committee at the College of William and Mary, which conforms to the guidelines of the US Public Health Service policy on humane care and use of laboratory animals (Office of Laboratory Animal Welfare, the National Institutes of Health, Bethesda, MD). To identify Dbx1-derived preBötC neurons, we crossed female tamoxifen-inducible Dbx1 Cre-driver mice (*Dbx1*^CreERT2^; Hirata *et al*., 2009; stock no. 028131, The Jackson Laboratory, Bar Harbor, ME) with male reporter mice whose *Rosa26* locus was modified by targeted insertion of a *loxP*-flanked STOP cassette followed by a gene for the fluorescent protein *tdTomato* (*Rosa26*^tdTomato^, stock no. 007905; The Jackson Laboratory). Tamoxifen (22.5 mg/kg body mass) was administered to pregnant dams during gestation at embryonic day 9.5 (ref. 16). In their offspring, *Dbx1*^CreERT2^; *Rosa26*^tdTomato^ mice, Cre-mediated recombination resulted in cytosolic tdTomato expression in cells derived from *Dbx1*-expressing progenitors.

### Medullary slices

Neonatal *Dbx1*^CreERT2^; *Rosa26*^tdTomato^ mice (postnatal day 2) of both sexes were anesthetized by hypothermia and their neuraxes were dissected in ice-cold artificial cerebrospinal fluid (aCSF) containing the following (in mM): 124 NaCl, 3 KCl, 1.5 CaCl_2_, 1 MgSO_4_, 25 NaHCO_3_, 0.5 NaH_2_PO_4_, and 30 dextrose equilibrated with 95% O_2_/5% CO_2_, pH 7.4. We prepared transverse brainstem sections (500 µm thick) whose rostral surface exposed the preBötC, which was verified by its position between the semi-compact division of the nucleus ambiguus and the dorsal boundary of the principal loop of the inferior olive^118^ (Fig. 1a). Slices were perfused with ice-cold aCSF bubbled with 95% O_2_/5% CO_2_ at 5 ml/min.

### Neuron isolation

Dbx1 preBötC neurons were identified using epifluorescence and removed under bright-field imaging (specifically, a version of differential interference contrast microscopy called ‘Dodt’ by Zeiss) on a fixed-stage microscope. Non-Dbx1 neurons were identified as neuronal somata without tdTomato fluorescence. We fabricated and heat-sterilized micropipettes from borosilicate capillary glass (1.50 mm outer diameter, 0.86 mm inner diameter). Micropipettes were devoid of solution prior to the isolating cells. After forming a seal with the plasma membrane, we applied negative pressure to draw single neurons into the tip of the micropipette (Fig. 1b). Tips containing the sampled neurons were immediately broken in sterile RNase/DNase-free tubes submersed in liquid nitrogen until a total of 15 neurons were collected. Each sample of 15 neurons was collected from a different animal (Fig. 1c, n = 6). We also performed mock cellular isolations as a control, bringing micropipettes into contact with the slice surface but without collecting cells. In mock cell isolations, we drew the same amount of fluid as real experiments, and performed cDNA synthesis in exactly the same manner as the real samples (n = 3).

### cDNA synthesis

Nuclease-free water and lysis buffer with RNase inhibitor, from the SMART-Seq v4 ultra low input RNA kit for Sequencing (634889, Clontech, Mountain View, CA), were added to each tube to raise the volume to 10.5 μL. The contents were incubated with sonication for 5 min to lyse the neurons and release cytoplasmic RNA, and then transferred to a 0.2 mL RNase-free PCR tube. First strand cDNA was synthesized by performing reverse transcription in a thermal cycler (42 °C for 90 min, 70 °C for 10 min). Then these cDNAs were primed with the 3′ SMART-Seq CDS Primer IIA, and we used the SMART-Seq v4 Oligonucleotide for template switching at the 5′ end of the transcript. The cDNA was then amplified by LD-PCR from the SMART sequences introduced by 3′ SMART-Seq CDS Primer IIA and the SMART-Seq v4 Oligonucleotide in a heated thermal cycler (95 °C for 1 min, 17 cycles of 98 °C for 10 s, 65 °C for 30 s, 68 °C for 3 min; 72 °C for 10 min). PCR-amplified cDNA was purified by immobilization on Agencourt AMPure XP beads (A63880, Beckman Coulter, Brea, CA), which were then washed with 80% ethanol and cDNA was eluted with elution buffer. Amplified cDNA was validated using the Agilent 2100 Bioanalyzer and Agilent’s High Sensitivity Kit (5067-4626, Agilent Technologies, Santa Clara, CA), and its concentration was determined using Qubit dsDNA High-sensitivity Assay Kit (Molecular Probes). The full-length cDNA output was processed with the Nextera Library Preparation Kits (FC-131-1024, Illumina, San Diego, CA) to obtain cDNA libraries for RNA-Seq experiments. Dbx1 and non-Dbx1 samples contained an average of 1481 ± 352 pg/µl of amplified cDNA, whereas mock cell-isolation samples contained 93 pg/µl cDNA (n = 3). Thus our transcriptome reflects cytoplasmic RNA from preBötC neurons. Figure 1d illustrates cDNA synthesis and quality control steps.

### Sequence analysis

The six cDNA libraries were submitted to the DNA Sequencing Center at Brigham Young University (Provo, UT) for sequencing on an Illumina - HiSeq. 2500. We received an average of 15,310,365 single-end reads per sample, with an average read length of 49.5 bp (range: 35–50 bp). We received these sequences and quality scores in the form of FASTQ files (first part of Fig. 1e) and aligned them to the mm10 murine genomic database from the University of California at Santa Cruz (http://hgdownload.soe.ucsc.edu/downloads.html) using Tophat2 software^119^ with the parameters specified in “TophatParameters.txt”, running on the Galaxy cluster computer at Johns Hopkins University (https://usegalaxy.org/). Average mapping rate was 85%. We sorted the binary alignment/map (BAM) files using Samtools (http://www.htslib.org/download/), utilities that process short DNA sequence read alignments, and then we applied HTSeq-count^120^ to map reads to genes with the following general command:

htseq-count -f ba×m -s no -i gene_name BAMFILE.bam genes.gtf> BAMFILE.txt

where genes.gtf is the gene annotation from the *Ensembl* (described below). This resulted in an average of 21,131,831 aligned reads across the six samples of which 7,815,666 uniquely mapped to genes. We implemented custom Python scripts to compute reads per kilobase of transcript per million mapped reads (RPKM) for each gene in each sample by normalizing for exon length according to the *Ensembl* mouse gene annotation database. These scripts read the *Ensembl* mouse gene annotation database (http://www.ensembl.org/Mus_musculus/Info/Index), and then apply the gtf-to-genes−1.40 package (https://pypi.python.org/pypi/gtf_to_genes) to extract the total exon length for each gene. We analysed transcription factors by cross-referencing our transcriptome data to the Riken Institute (Wako, Saitama, Japan) list of transcription factors (http://genome.gsc.riken.jp/TFdb/tf_list.html) and the TF-checkpoint database (http://www.tfcheckpoint.org/data/TFCheckpoint_download_180515.txt) from the Norwegian University of Science and Technology (Trondheim, Norway). We evaluated differential gene expression between Dbx1 and non-Dbx1 samples via the DESeq. 2^121^ algorithms performed on the HTSeq-count output (Fig. 1e).

We wrote more than 20 custom Python/R scripts to process the data displayed in Figs. 2–4 and Supplementary Figs S1–S9; and to process the DESeq. 2 results. These start from the BAM files generated from running the original FASTQ files on Tophat with the default parameters using the UCSC mm10 standard murine genome. They may be run sequentially from the beginning using the “runAll.sh” shell script that has been provided. The bioinformatics procedures are illustrated schematically in Fig. 1e. These scripts are freely available and open source (http://dbx1seq.sourceforge.net/).

### Differential expression

We evaluated differential gene expression between Dbx1 and non-Dbx1 neurons using DESeq. 2 (ref. 121), which computes the probability of obtaining the observed mean difference in gene expression if the Dbx1 and non-Dbx1 samples were drawn from the same underlying genetically homogenous population (the null hypothesis) as well as an adjusted probability, which reflects the false discovery rate (FDR) associated with multiple comparisons (*i.e*., ~50,000 genes). Significance level was set at FDR = 0.1 by convention. DESeq. 2 modifies the dataset to account for heteroscedasticity intrinsic to RNA-Seq data when calculating the raw p-value from a Wald test. DESeq. 2 calculates FDR^32^ using a Benjamini-Hochberg correction^31^, which aggressively combats Type I errors, *i.e*., false positive discoveries^31, 32^ at the expense of inflating false negatives (Type II errors)^33^. Acknowledging that p-value thresholds can be misleading if experiments are not analysed in context^122^, we interpret our data according to raw and adjusted probability (*i.e*., FDR) for genes that prior and corroborating literature suggests have respiratory neurobiological relevance. The associated log_2_ fold change (L2FC) quantifies the degree of differential expression. L2FC > 0 for genes more highly expressed in the Dbx1 samples; L2FC < 0 for genes more highly expressed in the non-Dbx1 samples.

### Data availability

The original data, which includes FASTQ files (raw nucleotide sequences and quality scores) and processed data files (sequenced reads that have been aligned and normalized to a murine reference genome) are publicly available in the NCBI Gene Expression Omnibus database, https://www.ncbi.nlm.nih.gov/geo/, accession number GSE100356.

## Electronic supplementary material

Supplementary Information
