# Supplementary material for: Transcriptome of neonatal preBötzinger complex neurones in Dbx1 reporter mice
Source: Sci Rep. 2017 Aug 17;7:8669. doi: 10.1038/s41598-017-09418-4 (PMC5561182; doi:10.1038/s41598-017-09418-4)
Supplement: Supplementary file 1 — Supplementary Information [file 41598_2017_9418_MOESM1_ESM.pdf]

Manuscript title: Transcriptome of neonatal preBötzinger complex neurones in Dbx1 reporter mice

Authors: John A. Hayes, Andrew Kottick; Maria Cristina D. Picardo; Andrew D. Halleran; Ronald D. Smith; Gregory D. Smith; Margaret S. Saha; and Christopher A. Del Negro

SUPPLEMENTARY INFORMATION

Charge carriers: inward currents

Sodium, calcium, and transient receptor potential (TRP) ion channels underlie inward currents in preBötC neurons.

Inspiratory bursts in preBötC neurons depend on periodic synaptic drive from the network of synaptically interconnected rhythmogenic neurons, as well as intrinsic inward charge carriers, including persistent Na^+ current (I_{NaP})[1–6] and Ca^{2+}-activated nonspecific cationic current (I_{CAN})[7–12]. Voltage-gated Ca^{2+} currents do not appear to be major charge carriers but rather mediate synaptic transmission and activate other currents (*e.g.*, Ca^{2+}-dependent K^+ currents and I_{CAN}) throughout the respiratory medulla, including the preBötC[13–16].

Na^+ currents arise from pore-forming α-subunits while β-subunits modulate voltage dependence and kinetics. Na_V 1.6 α-subunits give rise to persistent (I_{NaP}) and resurgent (I_{NaR}) Na^+ currents[17–19]. The β4 subunit, when co-expressed with the Na_V 1.6 α-subunit, produces I_{NaR} in particular[20].

In Dbx1 neurons, we found non-zero expression (Supplementary Fig. S3) of the pore-forming subunits Na_V1.1 (*Scn1a*), Na_V 1.2 (*Scn2a*), Na_V 1.3 (*Scn3a*), Na_V 1.6 (*Scn8a*), and Na_V 1.7 (*Scn9a*), which is broadly consistent with RT-PCR results obtained from rat preBötC neurons[4]. We also detected Na^+ channel-related β-subunits *Scn4b* and *Scn2b*. Between the Dbx1 and non-Dbx1 neurons, there was no differential expression between any of these sodium α- or β-subunits ($p > 0.05$ and $FDR > 0.1$).

The highest expression of Na^+ channel-related genes in either the Dbx1 or non-Dbx1 sample sets were the acid-sensing ion channels *Asic2* and *Asic1* (Supplementary Fig. S3), which is consistent with a recent report that both are widespread in ventrolateral medullary neurons[21].

A widely disseminated preBötC model proposed that a Na^+ leak current maintained high excitability in preBötC neurons[1,22]. The *Nalcn* gene codes for the underlying Na^+ leak channel, which is highly expressed in both Dbx1 and non-Dbx1 neurons (Supplementary Fig. S3). *Nalcn* is essential for normal breathing rhythms[23] and the NALCN channel is modulated by SP[24–26].

The role of I_{CAN} has not been unambiguously evaluated in living animals (*q.v.*, ref. 27), partially because its underlying ion channels are unknown, and because of promiscuous pharmacology[28]. It remains likely that I_{CAN} is mediated by a channel (or channels) from the transient receptor (TRP) superfamily[29–32]. TRP channels are quaternary and generally homomeric. Our group and Mironov hypothesized that I_{CAN} may be principally formed by TRPM4 or TRPM5 channels[10–12,33] because of their modulation by Ca^{2+} and monovalent cation permeability[29–32].

We found *Trp* genes expressed in Dbx1 preBötC neurons (Supplementary Fig. S4). *Trpc3* is particularly highly expressed (Dbx1 neurons RPKM = 18.5 ± 8.9). TRPC3 is therefore a novel candidate to exploit in transgenic *in vivo* studies aiming to address the source and importance of I_{CAN} in breathing behavior.

Trpc4 was differentially expressed between Dbx1 and non-Dbx1 neurons ($p = 0.00016$, FDR = 0.03) and higher in Dbx1 neurons specifically (L2FC = 2.30). It seems unlikely that TRPC4 gives rise to I_{CAN} in the preBötC because that channel typically forms receptor-operated Ca^{2+}-permeable cation channels[34–36] rather than Ca^{2+}-activated monovalent-permeable channels. It remains possible that *Trpc4* expression influences respiratory rhythm in a yet-to-be-determined manner, but because *Trpc4* homozygotic knockout mice breathe normally and are viable we think that unlikely[37].

Trpm4 was detected in both Dbx1 and non-Dbx1 neurons, but at relatively low levels compared to *Trpc3* and *Trpmc4* (Supplementary Fig. S4). *Trpm5* was completely undetectable in Dbx1 and non-Dbx1 neurons.

A balance of P/Q-type calcium channels (*Cacna1a*) and N-type calcium channels (*Cacna1b*) are required for stable respiratory activity within the preBötC[14], and indeed, we confirmed widespread expression of both classes in Dbx1 and non-Dbx1 neurons as (Supplementary Fig. S5). The three known Ca_V 3.X genes that give rise to T-type calcium channels (*Cacna1g*, *Cacna1h*, *Cacna1i*) were relatively sparsely expressed. Of the four generally recognized genes giving rise to L-type currents (*i.e.*, *Cacna1s* [Ca_V1.1], *Cacna1c* [Ca_V1.2], *Cacna1d* [Ca_V1.3], and *Cacna1f* [Ca_V1.4])[38], the only gene with non-negligible expression in either Dbx1 or non-Dbx1 neurons was the Ca_V1.3 (*Cacna1d*, RPKM = 3.53 [Dbx1 samples]). The R-type calcium channel mediating gene expression (*Cacna1e* [Ca_V2.3] was also moderately expressed in Dbx1 neurons (RPKM = 1.8).

Charge carriers: outward currents

Only a few studies have examined outward currents in preBötC neurons, such as activity-dependent outward currents involved in burst termination[39,40] and transient K^+ (I_A)[41–44].

Potassium currents are attributable to voltage-gated K^+ channels (KvX.X [*i.e.*, *KcnaX*, *KcnbX*, *KcncX*, *KcndX*, and *KcnhX*]), inward-rectifier K^+ channels (KirX.X [*i.e.*, *KcnjX*]), as well as Ca^{2+}-activated K^+ channels of the SK-type (K_{Ca}2.1 [*Kcnn1*], K_{Ca}2.2 [*Kcnn2*], and K_{Ca}2.3 [*Kcnn3*]), BK-type (K_{Ca}1.1 [*Kcnma1*]), and IK-type (KCa3.1 [*Kcnn4*]). Their expression levels are plotted in Supplementary Fig. S6.

The only K^+ current uniquely found in inspiratory neurons is I_A[39,44,45]. Inspiratory neurons that express I_A are among the earliest neurons to activate during a respiratory cycle[44,45], which is a sign of rhythmogenic function. We showed that 56% of Dbx1 preBötC neurons express I_A[46], which is consistent with the fraction of putatively rhythmogenic preBötC interneurons – classified as such based membrane properties and peptide

receptor expression – that express I_A[42,44]. In contrast only ~25% of non-Dbx1 neurons in the preBötC express I_A[43]. *Kcnd2* (K_V 4.2) was expressed in Dbx1 and non-Dbx1 neurons (Supplementary Fig. S6), although it was not differentially expressed (L2FC = 0.79, p = 0.045, FDR = 0.67). *Kcnd2* showed the second highest RPKM (23.5 ± 1.3) of any K^+-related genes, and the highest of all α-subunits, which suggests K_V 4.2 may still underlie I_A, although that remains to be tested.

We also identified a class of Ca^{2+}-binding K_V 4-interacting proteins (KChIPs) that influence channel density and kinetics of I_A[47–50]. KChIP2 (*Kcnip2*) was notable for its high expression in Dbx1 neurons (RPKM = 27.8 ± 12.9), as was and KChIP4 (*Kcnip4*, RPKM = 15.5 ± 6.3), but neither was differentially expressed by DESeq2 analyses ($L2FC_{Kcnip2}$ = 1.62, p = 0.0083, FDR = 0.32; $L2FC_{Kcnip4}$ = 1.77, p = 0.0016, FDR = 0.14). KChIP2 is thought to traffic K_V4.2 to the cell membrane[47] while KChIP4 likely gives K_V4.2 the slower inactivation kinetics[51] characteristic of I_A in preBötC neurons[45]. Those roles could apply to both Dbx1 and non-Dbx1 preBötC neurons.

The most highly expressed delayed-rectifier α-subunit in Dbx1 samples was *Kcnc4* (K_V3.4, RPKM = 19.0 ± 10.7), which was commensurate in Dbx1 and non-Dbx1 neurons (L2FC = 0.07, p = 0.91, FDR = 1.0).

The sub-threshold voltage-activated M-current is encoded by *Kcnq2* and *Kcnq3* (ref. 52). *Kcnq2* was the highest expressed gene in the class of non-transient and non-delayed-rectifier specifying potassium channels in Dbx1 neurons (Supplementary Fig. S6, middle block). KCNQ channels are associated with respiratory chemoreception[53]. It remains possible that KCNQ channels have some role in rhythmogenic preBötC microcircuits too.

preBötC neurons express hyperpolarization-activated mixed cation channels[54,55], and depending on the age, the blockade of this inward current can either speed up[55] or slow down[56] the respiratory rhythm. We detected all four *HcnX* channel genes in Dbx1 neurons (Supplementary Fig. S6), but there was no differential expression (all p > 0.05, FDR > 0.1).

Intracellular proteins

We proposed that inositol 1,4,5-trisphosphate receptors (IP_3R) contribute to intracellular Ca^{2+} release and I_{CAN} activation[10,11]. Three IP_3R subtypes (*Itpr1*, *Itpr2*, and *Itpr3*) showed low expression in Dbx1 neurons (combining *ItprX* gene expression together, RPKM = 0.3 ± 0.5, Supplementary Fig. S7). Intracellular Ca^{2+} release via ryanodine receptors (*Ryr1*, *Ryr2*, and *Ryr3*) could activate I_{CAN} as well. The *RyrX* transcripts were detected in Dbx1 and non-Dbx1 neurons (combining *RyrX* gene expression together, RPKM = 2.6 ± 2.1). *RyrXs* may be involved to some extent in sigh burst generation[56].

Pumps and exchangers

The Na/K ATPase pump has been associated with rhythmogenic functions in preBötC neurons, specifically inspiratory burst termination[39,40,57]. Isoforms of the α-subunit of the pump (α1, α2, α3) are expressed in the nervous system[58,59]. The α2 isoform is important for regular respiratory rhythm in the preBötC; α2-knockout mice are not viable[60,61]. We detected all four transcripts for the isoforms of Na/ATPase (Supplementary Fig. S7) but there was no differential expression. We also detected isoforms of the Na/Ca exchanger (*Slc8a1* and *Slc8a2*), among other pumps and exchangers (Supplementary Fig. S7).

Gap-junction-related proteins

preBötC neurons communicate via electrical synapses via gap junctions[62,63], which may influence how rhythm-generating neurons synchronize because neuropeptide excitation can be conveyed through gap junctions[24]. Both connexin 26 (*Gjb2*) and 32 (*Gjb1*) have been reported in the rat preBötC[63], and we detected those transcripts at relatively low levels (Supplementary Fig. S8). We detected connexins 37, 36, 43, and a number of others at relatively higher levels compared to connexins 26 and 32 (Supplementary Fig. S8).

Pannexins are a related class of non-junctional pore-forming channels that can release large molecules like ATP[64]. Pannexin 1 (*Panx1*) had the highest expression of the gap-

junction-related proteins in Dbx1 samples (Supplementary Fig. S8), but there was no evidence for differential expression (L2FC = -0.52, p = 0.07, FDR = 0.80).

Neuromodulation-related receptor transcripts

We examined gene transcripts for receptors for peptide, purine, and monoamine transmitters (Supplementary Fig. S9). Surprisingly, the peptide receptors in the preBötC that trigger sighs[65], *Nmbr* and *Grpr*, were ostensibly absent in Dbx1 neurons (RPKM_{Nmbr} = 0.02 ± 0.02, RPKM_{Grpr} = 0.0 ± 0.0) and were barely detectable in the non-Dbx1 samples (RPKM_{Nmbr} = 0.8 ± 0.6; RPKM_{Grpr} = 2.2 ± 2.2,). There was no evidence for differential expression (L2FC_{Nmbr} = -0.67, p = 0.20, FDR = 1.0; L2FC_{Grpr} = 0.0, p = 1.0, FDR = 1.0).

There was low purinergic receptor expression across Dbx1 neurons (combining all purinergic transcripts in Dbx1 neurons, RPKM = 0.9 ± 0.7) and non-Dbx1 neurons (combining all transcripts for non-Dbx1 neurons, RPKM = 1.7 ± 1.9). There was no evidence of differential expression (all p > 0.05, FDR > 0.1).

preBötC neurons receive serotonergic modulation from the raphé nucleus[66]. Manzke *et al.* (2003) demonstrated that serotonin can reduce the effects of opiate-induced respiratory depression specifically through 5-HT_{4A} (*i.e.*, *Htr4*) receptors[67], but those receptors were not differentially expressed (L2FC = -1.09, p = 0.09, FDR = 0.88). However, the transcript for the 5-HT_{1A} receptor (*Htr1a*) was differentially expressed, showing significantly greater expression in non-Dbx1 neurons (L2FC = -2.37, p = 0.0000314, FDR = 0.009). The 5-HT_{1A} receptor has been associated with glycinergic preBötC neurons[68]. Therefore, it is not surprising to find that 5-HT_{1A} receptor transcripts were associated with the non-Dbx1 neurons since Dbx1 neurons are predominantly glutamatergic.

Finally, we detected thyrotropin releasing hormone (TRH) receptors in Dbx1 and non-Dbx1 neurons, which did not meet the criterion for differential expression (L2FC_{Trhr} = 1.64, p = 0.011, FDR = 0.38). Nevertheless, in neonatal mice, TRH increases respiratory frequency *in vitro*, and excites the earliest activating preBötC neurons that

also express I_A and are putatively rhythmogenic[69]. In adult rats[41] The latter study showed that TRH excites preBötC neurons by blocking I_A. These data support the hypothesis that I_A-expressing Dbx1 neurons may be rhythmogenic.

SUPPLEMENTARY FIGURE LEGENDS

Figure S1. Heat maps showing expression levels for transcripts related to excitatory amino acid transmitter phenotype and excitatory amino acid receptors (**a**), or inhibitory amino acid transmitter phenotype and inhibitory amino acid receptors (**b**), sorted by highest mean RPKM in the Dbx1 samples. Genes at $p < 0.05$ are labelled in plain magenta typeface and those at FDR < 0.1 are labelled in bold magenta typeface if L2FC > 0. Genes at $p < 0.05$ are labelled in cyan typeface if L2FC < 0. RPKM is indicated by a pseudo-colour scale (right).

Figure S2. Heat maps showing relative levels of expression for gene transcripts related to peptide neurotransmitters and neuropeptide receptors, which are sorted by highest mean RPKM in the Dbx1 samples. *Tac1*, the only gene with L2FC > 0 and $p < 0.05$ is labelled in plain magenta typeface. RPKM is indicated by a pseudo-colour scale (right).

Figure S3. Heat map showing relative levels of expression for transcripts related to Na^+ channels, which are sorted by highest mean RPKM in the Dbx1 samples. RPKM is indicated by a pseudo-colour scale (right).

Figure S4. Heat map showing relative levels expression of the transcripts for TRP channels, which are sorted by highest mean RPKM in the Dbx1 samples. The gene for which L2FC > 0 and FDR < 0.1 is labelled in bold magenta typeface. RPKM is indicated by a pseudo-colour scale (right).

Figure S5. Heat map showing relative levels of expression for transcripts related to Ca^{2+} channels, which are sorted by highest mean RPKM in the Dbx1 samples. Genes at $p < 0.05$ for differential expression are labelled in plain magenta typeface if L2FC > 0, whereas if L2FC < 0, then the genes at $p < 0.05$ are labelled in plain cyan typeface. RPKM is indicated by a pseudo-colour scale (right).

Figure S6. Heat map showing relative levels of expression for transcripts related to K^+ channels, which are sorted by highest mean RPKM in the Dbx1 samples. Genes at $p < 0.05$ for differential expression are labelled in plain magenta typeface if L2FC > 0, whereas if L2FC < 0, then the genes at $p < 0.05$ are labelled in plain cyan typeface. RPKM is indicated by a pseudo-colour scale (right).

Figure S7. Heat maps showing relative levels of expression for intracellular Ca^{2+}-release-related receptors, ATPase pumps, and passive transport exchangers. The data are sorted by highest mean RPKM in the Dbx1 samples. RPKM is indicated by a pseudo-colour scale (right).

Figure S8. Heat map showing relative levels of expression for connexin and pan nexin proteins related to gap junctions. Data are sorted by highest mean RPKM in the Dbx1 samples. RPKM is indicated by a pseudo-colour scale (right).

Figure S9. Heat maps showing relative levels of expression for neuromodulators identified by physiological studies as being relevant to respiratory neural control. Data are sorted by highest mean RPKM in the Dbx1 samples. Genes at $p < 0.05$ for differential expression are labelled in plain magenta typeface if L2FC > 0, whereas for L2FC < 0 the gene at FDR < 0.1 is labelled in bold cyan typeface. RPKM is indicated by a pseudo-colour scale (right).

REFERENCES

1. Del Negro, C. A., Johnson, S. M., But era, R. J. & Smith, J. C. Models of respiratory rhythm generation in the pre-Bötzinger complex. III. Experimental tests of model predictions. *J. Neurophysiol.* **86,** 59–74 (2001).
2. Del Negro, C. A., Koshiya, N., Butera, R. J., Jr & Smith, J. C. Persistent sodium current, membrane properties and bursting behavior of pre-bötzinger complex inspiratory neurons in vitro. *J. Neurophysiol.* **88,** 2242–2250 (2002).
3. Koizumi, H. & Smith, J. C. Persistent Na+ and K+-dominated leak currents contribute to respiratory rhythm generation in the pre-Bötzinger complex in vitro. *J. Neurosci. Off. J. Soc. Neurosci.* **28,** 1773–1785 (2008).
4. Ptak, K. *et al.* Sodium currents in medullary neurons isolated from the pre-Bötzinger complex region. *J. Neurosci.* **25,** 5159–5170 (2005).
5. Pace, R. W., Mackay, D. D., Feldman, J. L. & Del Negro, C. A. Role of persistent sodium current in mouse preBötzinger Complex neurons and respiratory rhythm generation. *J. Physiol.* **580,** 485–496 (2007).

6. Del Negro, C. A., Morgado-Valle, C. & Feldman, J. L. Respiratory rhythm: an emergent network property? *Neuron* **34,** 821–830 (2002).
7. Thoby-Brisson, M. & Ramirez, J. M. Identification of two types of inspiratory pacemaker neurons in the isolated respiratory neural network of mice. *J. Neurophysiol.* **86,** 104–112 (2001).
8. Peña, F., Parkis, M. A., Tryba, A. K. & Ramirez, J.-M. Differential contribution of pacemaker properties to the generation of respiratory rhythms during normoxia and hypoxia. *Neuron* **43,** 105–117 (2004).
9. Del Negro, C. A. *et al.* Sodium and calcium current-mediated pacemaker neurons and respiratory rhythm generation. *J. Neurosci. Off. J. Soc. Neurosci.* **25,** 446–453 (2005).
10. Crowder, E. A. *et al.* Phosphatidylinositol 4,5-bisphosphate regulates inspiratory burst activity in the neonatal mouse preBötzinger complex. *J. Physiol.* **582,** 1047–1058 (2007).
11. Pace, R. W., Mackay, D. D., Feldman, J. L. & Del Negro, C. A. Inspiratory bursts in the preBötzinger complex depend on a calcium-activated non-specific cation current linked to glutamate receptors in neonatal mice. *J. Physiol.* **582,** 113–125 (2007).
12. Mironov, S. L. Metabotropic glutamate receptors activate dendritic calcium waves and TRPM channels which drive rhythmic respiratory patterns in mice. *J. Physiol.* **586,** 2277–2291 (2008).
13. Elsen, F. P. & Ramirez, J. M. Calcium currents of rhythmic neurons recorded in the isolated respiratory network of neonatal mice. *J. Neurosci. Off. J. Soc. Neurosci.* **18,** 10652–10662 (1998).
14. Koch, H. *et al.* Stable Respiratory Activity Requires Both P/Q-Type and N-Type Voltage-Gated Calcium Channels. *J. Neurosci.* **33,** 3633–3645 (2013).
15. Pierrefiche, O., Champagnat, J. & Richter, D. W. Calcium-dependent conductances control neurones involved in termination of inspiration in cats. *Neurosci. Lett.* **184,** 101–104 (1995).
16. Onimaru, H., Ballanyi, K. & Homma, I. Contribution of Ca2+-dependent conductances to membrane potential fluctuations of medullary respiratory neurons of newborn rats in vitro. *J. Physiol.* **552,** 727–741 (2003).
17. Osorio, N. *et al.* Persistent Nav1.6 current at axon initial segments tunes spike timing of cerebellar granule cells. *J. Physiol.* **588,** 651–670 (2010).
18. Aman, T. K. *et al.* Regulation of Persistent Na Current by Interactions between Subunits of Voltage-Gated Na Channels. *J. Neurosci.* **29,** 2027–2042 (2009).
19. Raman, I. M., Sprunger, L. K., Meisler, M. H. & Bean, B. P. Altered Subthreshold Sodium Currents and Disrupted Firing Patterns in Purkinje Neurons of Scn8a Mutant Mice. *Neuron* **19,** 881–891 (1997).
20. Grieco, T. M., Malhotra, J. D., Chen, C., Isom, L. L. & Raman, I. M. Open-Channel Block by the Cytoplasmic Tail of Sodium Channel β4 as a Mechanism for Resurgent Sodium Current. *Neuron* **45,** 233–244 (2005).
21. Song, N. *et al.* Acid-sensing ion channels are expressed in the ventrolateral medulla and contribute to central chemoreception. *Sci. Rep.* **6,** (2016).

22. Butera, R. J., Rinzel, J. & Smith, J. C. Models of respiratory rhythm generation in the pre-Bötzinger complex. I. Bursting pacemaker neurons. *J. Neurophysiol.* **82,** 382–397 (1999).
23. Lu, B. *et al.* The neuronal channel NALCN contributes resting sodium permeability and is required for normal respiratory rhythm. *Cell* **129,** 371–383 (2007).
24. Hayes, J. A. & Del Negro, C. A. Neurokinin receptor-expressing pre-botzinger complex neurons in neonatal mice studied in vitro. *J. Neurophysiol.* **97,** 4215–4224 (2007).
25. Lu, B. *et al.* Peptide neurotransmitters activate a cation channel complex of NALCN and UNC-80. *Nature* **457,** 741–744 (2009).
26. Yeh, S.-Y. *et al.* Respiratory Network Stability and Modulatory Response to Substance P Require Nalcn. *Neuron* **94,** 294–303.e4 (2017).
27. Peña, F. & Aguileta, M.-A. Effects of riluzole and flufenamic acid on eupnea and gasping of neonatal mice in vivo. *Neurosci. Lett.* **415,** 288–293 (2007).
28. Guinamard, R., Simard, C. & Del Negro, C. Flufenamic acid as an ion channel modulator. *Pharmacol. Ther.* **138,** 272–284 (2013).
29. Ullrich, N. D. *et al.* Comparison of functional properties of the Ca2+-activated cation channels TRPM4 and TRPM5 from mice. *Cell Calcium* **37,** 267–278 (2005).
30. Owsianik, G., Talavera, K., Voets, T. & Nilius, B. PERMEATION AND SELECTIVITY OF TRP CHANNELS. *Annu. Rev. Physiol.* **68,** 685–717 (2006).
31. Hofmann, T., Chubanov, V., Gudermann, T. & Montell, C. TRPM5 Is a Voltage-Modulated and Ca2+-Activated Monovalent Selective Cation Channel. *Curr. Biol.* **13,** 1153–1158 (2003).
32. Launay, P. *et al.* TRPM4 Is a Ca2+-Activated Nonselective Cation Channel Mediating Cell Membrane Depolarization. *Cell* **109,** 397–407 (2002).
33. Mironov, S. L. & Skorova, E. Y. Stimulation of bursting in pre-Bötzinger neurons by Epac through calcium release and modulation of TRPM4 and K-ATP channels. *J. Neurochem.* **117,** 295–308 (2011).
34. Plant, T. D. & Schaefer, M. TRPC4 and TRPC5: receptor-operated Ca2+-permeable nonselective cation channels. *Cell Calcium* **33,** 441–450 (2003).
35. Schaefer, M. *et al.* Receptor-mediated Regulation of the Nonselective Cation Channels TRPC4 and TRPC5. *J. Biol. Chem.* **275,** 17517–17526 (2000).
36. Thakur, D. P. *et al.* Critical roles of Gi/o proteins and phospholipase C-δ1 in the activation of receptor-operated TRPC4 channels. *Proc. Natl. Acad. Sci. U. S. A.* **113,** 1092–1097 (2016).
37. Westlund, K. N. *et al.* A rat knockout model implicates TRPC4 in visceral pain sensation. *Neuroscience* **262,** 165–175 (2014).
38. Catterall, W. A., Perez-Reyes, E., Snutch, T. P. & Striessnig, J. International Union of Pharmacology. XLVIII. Nomenclature and Structure-Function Relationships of Voltage-Gated Calcium Channels. *Pharmacol. Rev.* **57,** 411–425 (2005).
39. Krey, R. A., Goodreau, A. M., Arnold, T. B. & Del Negro, C. A. Outward Currents Contributing to Inspiratory Burst Termination in preBötzinger Complex Neurons of Neonatal Mice Studied in Vitro. *Front. Neural Circuits* **4,** 124 (2010).
40. Del Negro, C. A., Kam, K., Hayes, J. A. & Feldman, J. L. Asymmetric control of inspiratory and expiratory phases by excitability in the respiratory network of neonatal mice in vitro. *J. Physiol.* **587,** 1217–1231 (2009).

41. Inyushkin, A. N. Thyroliberin blocks the potassium A-current in neurons in the respiratory center of adult rats in vitro. *Neurosci. Behav. Physiol.* **35,** 549–554 (2005).

42. Hayes, J. A., Mendenhall, J. L., Brush, B. R. & Del Negro, C. A. 4-Aminopyridine-sensitive outward currents in preBötzinger complex neurons influence respiratory rhythm generation in neonatal mice. *J. Physiol.* **586,** 1921–1936 (2008).

43. Picardo, M. C. D., Weragalaarachchi, K. T. H., Akins, V. T. & Del Negro, C. A. Physiological and morphological properties of Dbx1-derived respiratory neurons in the pre-Bötzinger complex of neonatal mice. *J. Physiol.* **591,** 2687–2703 (2013).

44. Rekling, J. C., Champagnat, J. & Denavit-Saubié, M. Electroresponsive properties and membrane potential trajectories of three types of inspiratory neurons in the newborn mouse brain stem in vitro. *J. Neurophysiol.* **75,** 795–810 (1996).

45. Hayes, J. A., Mendenhall, J. L., Brush, B. R. & Del Negro, C. A. 4-Aminopyridine-sensitive outward currents in preBötzinger complex neurons influence respiratory rhythm generation in neonatal mice. *J. Physiol.* **586,** 1921–1936 (2008).

46. Picardo, M. C. D., Weragalaarachchi, K. T. H., Akins, V. T. & Del Negro, C. A. Physiological and morphological properties of Dbx1-derived respiratory neurons in the pre-Bötzinger complex of neonatal mice. *J. Physiol.* **591,** 2687–2703 (2013).

47. Shibata, R. *et al.* A Fundamental Role for KChIPs in Determining the Molecular Properties and Trafficking of Kv4.2 Potassium Channels. *J. Biol. Chem.* **278,** 36445–36454 (2003).

48. An, W. F. *et al.* Modulation of A-type potassium channels by a family of calcium sensors. *Nature* **403,** 553–556 (2000).

49. Rhodes, K. J. *et al.* KChIPs and Kv4 α Subunits as Integral Components of A-Type Potassium Channels in Mammalian Brain. *J. Neurosci.* **24,** 7903–7915 (2004).

50. Wang, H. *et al.* Structural basis for modulation of Kv4 K+ channels by auxiliary KChIP subunits. *Nat. Neurosci.* **10,** 32–39 (2007).

51. Baranauskas, G. Cell-type-specific splicing of KChIP4 mRNA correlates with slower kinetics of A-type current. *Eur. J. Neurosci.* **20,** 385–391 (2004).

52. Wang, H.-S. *et al.* KCNQ2 and KCNQ3 Potassium Channel Subunits: Molecular Correlates of the M-Channel. *Science* **282,** 1890–1893 (1998).

53. Mulkey, D. K. *et al.* Molecular underpinnings of ventral surface chemoreceptor function: focus on KCNQ channels. *J. Physiol.* **593,** 1075–1081 (2015).

54. Mironov, S. L., Langohr, K. & Richter, D. W. Hyperpolarization-activated current, Ih, in inspiratory brainstem neurons and its inhibition by hypoxia. *Eur. J. Neurosci.* **12,** 520–526 (2000).

55. Thoby-Brisson, M., Telgkamp, P. & Ramirez, J. M. The role of the hyperpolarization-activated current in modulating rhythmic activity in the isolated respiratory network of mice. *J. Neurosci. Off. J. Soc. Neurosci.* **20,** 2994–3005 (2000).

56. Toporikova, N., Chevalier, M. & Thoby-Brisson, M. Sigh and Eupnea Rhythmogenesis Involve Distinct Interconnected Subpopulations: A Combined Computational and Experimental Study(1,2,3). *eNeuro* **2,** (2015).

57. Rubin, J. E., Hayes, J. A., Mendenhall, J. L. & Del Negro, C. A. Calcium-activated nonspecific cation current and synaptic depression promote network-dependent burst oscillations. *Proc. Natl. Acad. Sci. U. S. A.* **106,** 2939–2944 (2009).

58. Lingrel, J. *et al.* Functional roles of the alpha isoforms of the Na,K-ATPase. *Ann. N. Y. Acad. Sci.* **986,** 354–359 (2003).
59. Herrera, V. L., Cova, T., Sassoon, D. & Ruiz-Opazo, N. Developmental cell-specific regulation of Na(+)-K(+)-ATPase alpha 1-, alpha 2-, and alpha 3-isoform gene expression. *Am. J. Physiol.* **266,** C1301-1312 (1994).
60. Ikeda, K. *et al.* Malfunction of respiratory-related neuronal activity in Na+, K+-ATPase alpha2 subunit-deficient mice is attributable to abnormal Cl- homeostasis in brainstem neurons. *J. Neurosci. Off. J. Soc. Neurosci.* **24,** 10693–10701 (2004).
61. Moseley, A. E. *et al.* The Na,K-ATPase alpha 2 isoform is expressed in neurons, and its absence disrupts neuronal activity in newborn mice. *J. Biol. Chem.* **278,** 5317–5324 (2003).
62. Rekling, J. C., Shao, X. M. & Feldman, J. L. Electrical coupling and excitatory synaptic transmission between rhythmogenic respiratory neurons in the preBötzinger complex. *J. Neurosci. Off. J. Soc. Neurosci.* **20,** RC113 (2000).
63. Solomon, I. C., Halat, T. J., El-Maghrabi, R. & O'Neal, M. H. Differential expression of connexin26 and connexin32 in the pre-Bötzinger complex of neonatal and adult rat. *J. Comp. Neurol.* **440,** 12–19 (2001).
64. MacVicar, B. A. & Thompson, R. J. Non-junction functions of pannexin-1 channels. *Trends Neurosci.* **33,** 93–102 (2010).
65. Li, P. *et al.* The peptidergic control circuit for sighing. *Nature* **530,** 293–297 (2016).
66. Ptak, K. *et al.* Raphé neurons stimulate respiratory circuit activity by multiple mechanisms via endogenously released serotonin and substance P. *J. Neurosci. Off. J. Soc. Neurosci.* **29,** 3720–3737 (2009).
67. Manzke, T. *et al.* 5-HT4(a) receptors avert opioid-induced breathing depression without loss of analgesia. *Science* **301,** 226–229 (2003).
68. Manzke, T. *et al.* Serotonin receptor 1A–modulated phosphorylation of glycine receptor α3 controls breathing in mice. *J. Clin. Invest.* **120,** 4118–4128 (2010).
69. Rekling, J. C., Champagnat, J. & Denavit-Saubié, M. Thyrotropin-releasing hormone (TRH) depolarizes a subset of inspiratory neurons in the newborn mouse brain stem in vitro. *J. Neurophysiol.* **75,** 811–819 (1996).

Figure S1

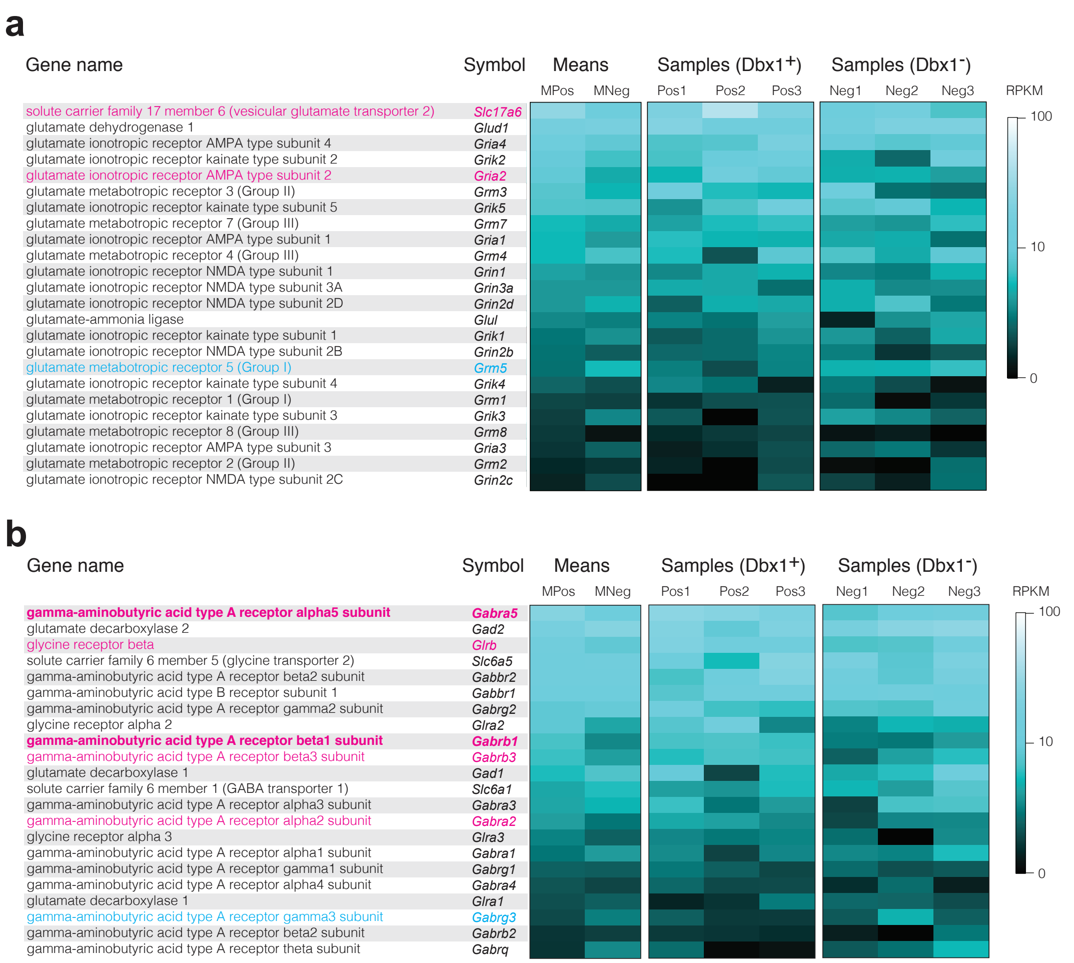
a
Gene name
Symbol
Means
Samples (Dbx1+)
Samples (Dbx1-)
MPos
MNeg
Pos1
Pos2
Pos3
Neg1
Neg2
Neg3
RPKM
100
10
0
solute carrier family 17 member 6 (vesicular glutamate transporter 2) Slc17a6
glutamate dehydrogenase 1 Glud1
glutamate ionotropic receptor AMPA type subunit 4 Gria4
glutamate ionotropic receptor kainate type subunit 2 Grik2
glutamate ionotropic receptor AMPA type subunit 2 Gria2
glutamate metabotropic receptor 3 (Group II) Grm3
glutamate ionotropic receptor kainate type subunit 5 Grik5
glutamate metabotropic receptor 7 (Group III) Grm7
glutamate ionotropic receptor AMPA type subunit 1 Gria1
glutamate metabotropic receptor 4 (Group III) Grm4
glutamate ionotropic receptor NMDA type subunit 1 Grin1
glutamate ionotropic receptor NMDA type subunit 3A Grin3a
glutamate ionotropic receptor NMDA type subunit 2D Grin2d
glutamate-ammonia ligase Glul
glutamate ionotropic receptor kainate type subunit 1 Grik1
glutamate ionotropic receptor NMDA type subunit 2B Grin2b
glutamate metabotropic receptor 5 (Group I) Grm5
glutamate ionotropic receptor kainate type subunit 4 Grik4
glutamate metabotropic receptor 1 (Group I) Grm1
glutamate ionotropic receptor kainate type subunit 3 Grik3
glutamate metabotropic receptor 8 (Group III) Grm8
glutamate ionotropic receptor AMPA type subunit 3 Gria3
glutamate metabotropic receptor 2 (Group II) Grm2
glutamate ionotropic receptor NMDA type subunit 2C Grin2c
b
Gene name
Symbol
Means
Samples (Dbx1+)
Samples (Dbx1-)
MPos
MNeg
Pos1
Pos2
Pos3
Neg1
Neg2
Neg3
RPKM
100
10
0
gamma-aminobutyric acid type A receptor alpha5 subunit Gabra5
glutamate decarboxylase 2 Gad2
glycine receptor beta Glrb
solute carrier family 6 member 5 (glycine transporter 2) Slc6a5
gamma-aminobutyric acid type A receptor beta2 subunit Gabbr2
gamma-aminobutyric acid type B receptor subunit 1 Gabbr1
gamma-aminobutyric acid type A receptor gamma2 subunit Gabrg2
glycine receptor alpha 2 Glra2
gamma-aminobutyric acid type A receptor beta1 subunit Gabrb1
gamma-aminobutyric acid type A receptor beta3 subunit Gabrb3
glutamate decarboxylase 1 Gad1
solute carrier family 6 member 1 (GABA transporter 1) Slc6a1
gamma-aminobutyric acid type A receptor alpha3 subunit Gabra3
gamma-aminobutyric acid type A receptor alpha2 subunit Gabra2
glycine receptor alpha 3 Glra3
gamma-aminobutyric acid type A receptor alpha1 subunit Gabra1
gamma-aminobutyric acid type A receptor gamma1 subunit Gabrg1
gamma-aminobutyric acid type A receptor alpha4 subunit Gabra4
glutamate decarboxylase 1 Glra1
gamma-aminobutyric acid type A receptor gamma3 subunit Gabrg3
gamma-aminobutyric acid type A receptor beta2 subunit Gabrb2
gamma-aminobutyric acid type A receptor theta subunit Gabrq

Figure S2

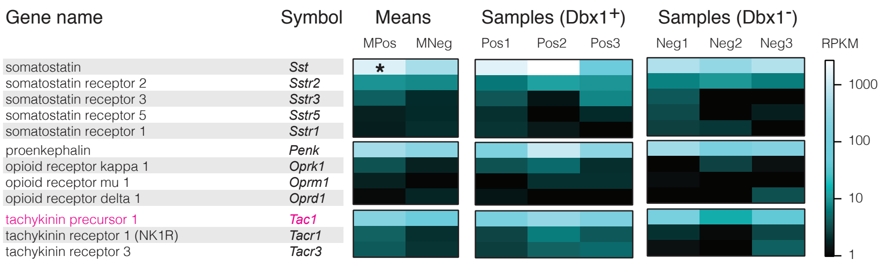
Gene name
Symbol
Means
Samples (Dbx1+)
Samples (Dbx1-)
MPos
MNeg
Pos1
Pos2
Pos3
Neg1
Neg2
Neg3
RPKM
somatostatin
somatostatin receptor 2
somatostatin receptor 3
somatostatin receptor 5
somatostatin receptor 1
proenkephalin
opioid receptor kappa 1
opioid receptor mu 1
opioid receptor delta 1
tachykinin precursor 1
tachykinin receptor 1 (NK1R)
tachykinin receptor 3
Sst
Sstr2
Sstr3
Sstr5
Sstr1
Penk
Oprk1
Oprm1
Oprd1
Tac1
Tacr1
Tacr3
*
1000
100
10
1

Figure S3

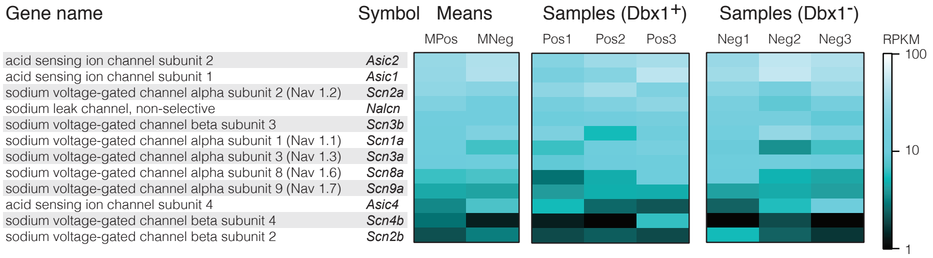
Gene name
Symbol
Means
Samples (Dbx1+)
Samples (Dbx1-)
MPos
MNeg
Pos1
Pos2
Pos3
Neg1
Neg2
Neg3
RPKM
100
10
1
acid sensing ion channel subunit 2
Asic2
acid sensing ion channel subunit 1
Asic1
sodium voltage-gated channel alpha subunit 2 (Nav 1.2)
Scn2a
sodium leak channel, non-selective
Nalcn
sodium voltage-gated channel beta subunit 3
Scn3b
sodium voltage-gated channel alpha subunit 1 (Nav 1.1)
Scn1a
sodium voltage-gated channel alpha subunit 3 (Nav 1.3)
Scn3a
sodium voltage-gated channel alpha subunit 8 (Nav 1.6)
Scn8a
sodium voltage-gated channel alpha subunit 9 (Nav 1.7)
Scn9a
acid sensing ion channel subunit 4
Asic4
sodium voltage-gated channel beta subunit 4
Scn4b
sodium voltage-gated channel beta subunit 2
Scn2b

Figure S4

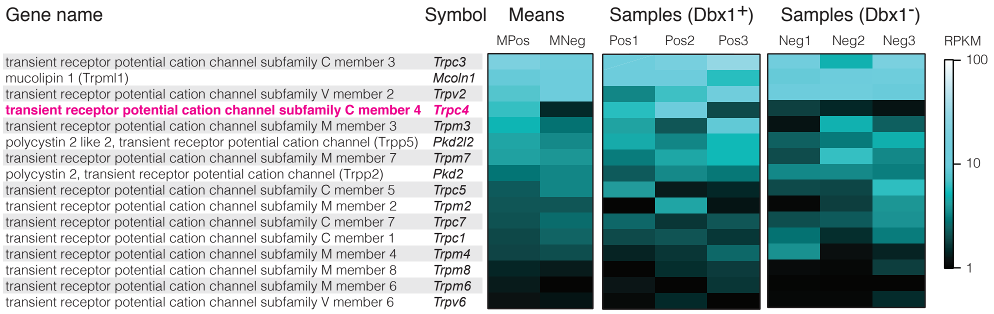
Gene name
Symbol
Means
Samples (Dbx1+)
Samples (Dbx1-)
MPos
MNeg
Pos1
Pos2
Pos3
Neg1
Neg2
Neg3
RPKM
100
10
1
transient receptor potential cation channel subfamily C member 3 Trpc3
mucolipin 1 (Trpml1) Mcoln1
transient receptor potential cation channel subfamily V member 2 Trpv2
transient receptor potential cation channel subfamily C member 4 Trpc4
transient receptor potential cation channel subfamily M member 3 Trpm3
polycystin 2 like 2, transient receptor potential cation channel (Trpp5) Pkd2l2
transient receptor potential cation channel subfamily M member 7 Trpm7
polycystin 2, transient receptor potential cation channel (Trpp2) Pkd2
transient receptor potential cation channel subfamily C member 5 Trpc5
transient receptor potential cation channel subfamily M member 2 Trpm2
transient receptor potential cation channel subfamily C member 7 Trpc7
transient receptor potential cation channel subfamily C member 1 Trpc1
transient receptor potential cation channel subfamily M member 4 Trpm4
transient receptor potential cation channel subfamily M member 8 Trpm8
transient receptor potential cation channel subfamily M member 6 Trpm6
transient receptor potential cation channel subfamily V member 6 Trpv6

Figure S5

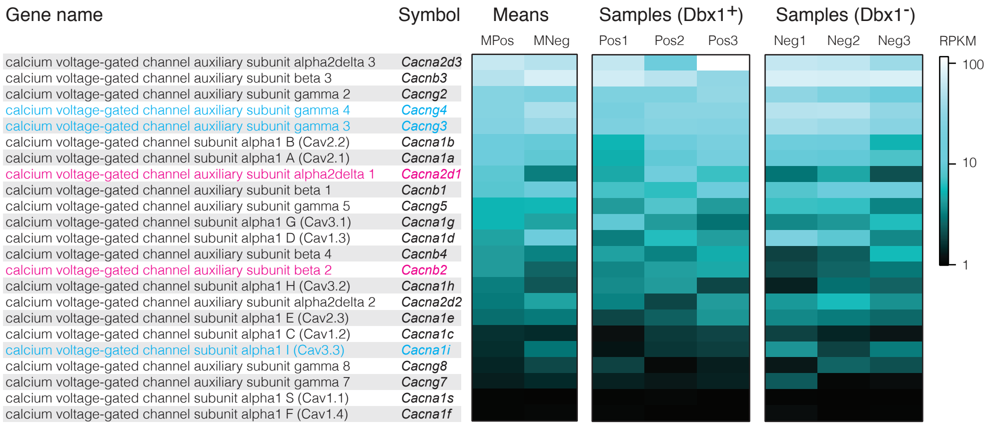

Gene name
Symbol
Means
Samples (Dbx1+)
Samples (Dbx1-)
MPos
MNeg
Pos1
Pos2
Pos3
Neg1
Neg2
Neg3
RPKM
100
10
1
calcium voltage-gated channel auxiliary subunit alpha2delta 3
Cacna2d3
calcium voltage-gated channel auxiliary subunit beta 3
Cacnb3
calcium voltage-gated channel auxiliary subunit gamma 2
Cacng2
calcium voltage-gated channel auxiliary subunit gamma 4
Cacng4
calcium voltage-gated channel auxiliary subunit gamma 3
Cacng3
calcium voltage-gated channel subunit alpha1 B (Cav2.2)
Cacna1b
calcium voltage-gated channel subunit alpha1 A (Cav2.1)
Cacna1a
calcium voltage-gated channel auxiliary subunit alpha2delta 1
Cacna2d1
calcium voltage-gated channel auxiliary subunit beta 1
Cacnb1
calcium voltage-gated channel auxiliary subunit gamma 5
Cacng5
calcium voltage-gated channel subunit alpha1 G (Cav3.1)
Cacna1g
calcium voltage-gated channel subunit alpha1 D (Cav1.3)
Cacna1d
calcium voltage-gated channel auxiliary subunit beta 4
Cacnb4
calcium voltage-gated channel auxiliary subunit beta 2
Cacnb2
calcium voltage-gated channel subunit alpha1 H (Cav3.2)
Cacna1h
calcium voltage-gated channel auxiliary subunit alpha2delta 2
Cacna2d2
calcium voltage-gated channel subunit alpha1 E (Cav2.3)
Cacna1e
calcium voltage-gated channel subunit alpha1 C (Cav1.2)
Cacna1c
calcium voltage-gated channel subunit alpha1 I (Cav3.3)
Cacna1i
calcium voltage-gated channel auxiliary subunit gamma 8
Cacng8
calcium voltage-gated channel auxiliary subunit gamma 7
Cacng7
calcium voltage-gated channel subunit alpha1 S (Cav1.1)
Cacna1s
calcium voltage-gated channel subunit alpha1 F (Cav1.4)
Cacna1f

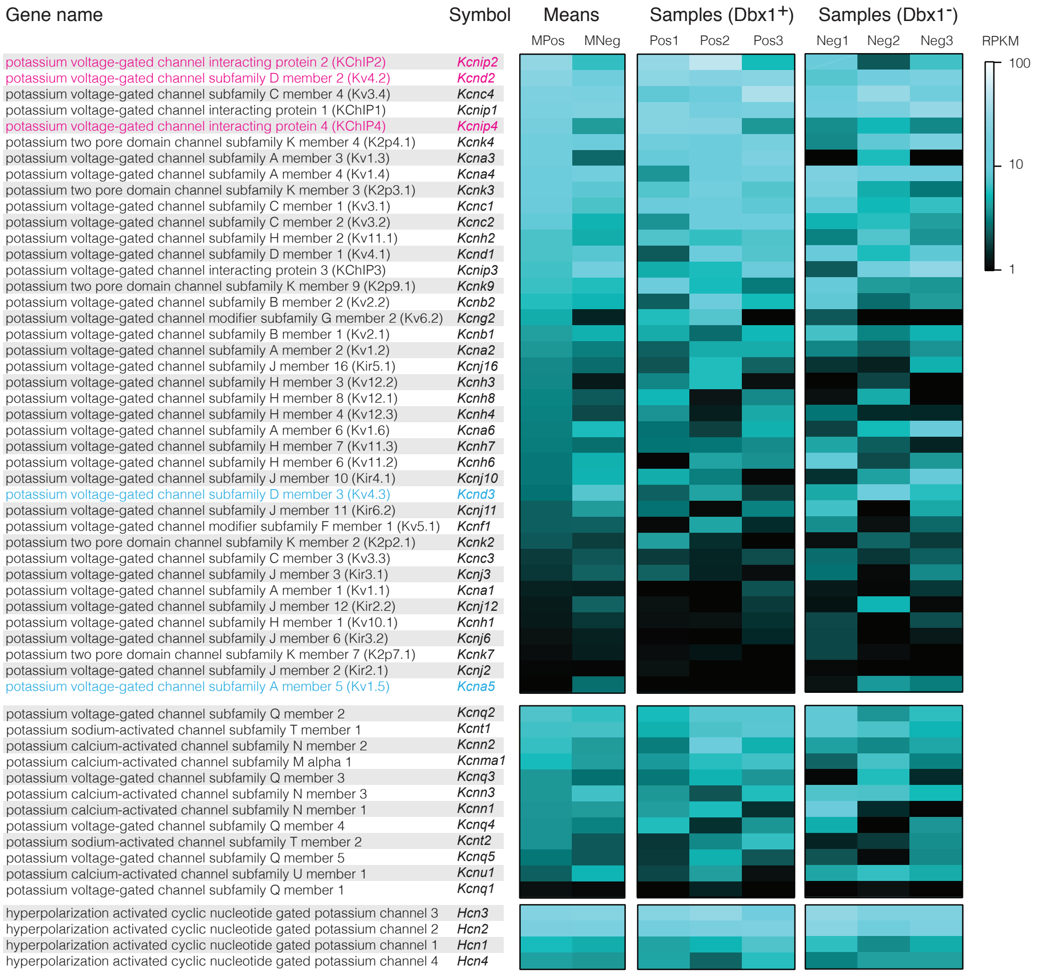
Gene name
Symbol
Means
Samples (Dbx1+)
Samples (Dbx1-)
MPos
MNeg
Pos1
Pos2
Pos3
Neg1
Neg2
Neg3
RPKM
100
10
1
potassium voltage-gated channel interacting protein 2 (KChIP2) Kcnip2
potassium voltage-gated channel subfamily D member 2 (Kv4.2) Kcnd2
potassium voltage-gated channel subfamily C member 4 (Kv3.4) Kcnc4
potassium voltage-gated channel interacting protein 1 (KChIP1) Kcnip1
potassium voltage-gated channel interacting protein 4 (KChIP4) Kcnip4
potassium two pore domain channel subfamily K member 4 (K2p4.1) Kcnk4
potassium voltage-gated channel subfamily A member 3 (Kv1.3) Kcna3
potassium voltage-gated channel subfamily A member 4 (Kv1.4) Kcna4
potassium two pore domain channel subfamily K member 3 (K2p3.1) Kcnk3
potassium voltage-gated channel subfamily C member 1 (Kv3.1) Kcnc1
potassium voltage-gated channel subfamily C member 2 (Kv3.2) Kcnc2
potassium voltage-gated channel subfamily H member 2 (Kv11.1) Kcnh2
potassium voltage-gated channel subfamily D member 1 (Kv4.1) Kcnd1
potassium voltage-gated channel interacting protein 3 (KChIP3) Kcnip3
potassium two pore domain channel subfamily K member 9 (K2p9.1) Kcnk9
potassium voltage-gated channel subfamily B member 2 (Kv2.2) Kcnb2
potassium voltage-gated channel modifier subfamily G member 2 (Kv6.2) Kcng2
potassium voltage-gated channel subfamily B member 1 (Kv2.1) Kcnb1
potassium voltage-gated channel subfamily A member 2 (Kv1.2) Kcna2
potassium voltage-gated channel subfamily J member 16 (Kir5.1) Kcnj16
potassium voltage-gated channel subfamily H member 3 (Kv12.2) Kcnh3
potassium voltage-gated channel subfamily H member 8 (Kv12.1) Kcnh8
potassium voltage-gated channel subfamily H member 4 (Kv12.3) Kcnh4
potassium voltage-gated channel subfamily A member 6 (Kv1.6) Kcna6
potassium voltage-gated channel subfamily H member 7 (Kv11.3) Kcnh7
potassium voltage-gated channel subfamily H member 6 (Kv11.2) Kcnh6
potassium voltage-gated channel subfamily J member 10 (Kir4.1) Kcnj10
potassium voltage-gated channel subfamily D member 3 (Kv4.3) Kcnd3
potassium voltage-gated channel subfamily J member 11 (Kir6.2) Kcnj11
potassium voltage-gated channel modifier subfamily F member 1 (Kv5.1) Kcnf1
potassium two pore domain channel subfamily K member 2 (K2p2.1) Kcnk2
potassium voltage-gated channel subfamily C member 3 (Kv3.3) Kcnc3
potassium voltage-gated channel subfamily J member 3 (Kir3.1) Kcnj3
potassium voltage-gated channel subfamily A member 1 (Kv1.1) Kcna1
potassium voltage-gated channel subfamily J member 12 (Kir2.2) Kcnj12
potassium voltage-gated channel subfamily H member 1 (Kv10.1) Kcnh1
potassium voltage-gated channel subfamily J member 6 (Kir3.2) Kcnj6
potassium two pore domain channel subfamily K member 7 (K2p7.1) Kcnk7
potassium voltage-gated channel subfamily J member 2 (Kir2.1) Kcnj2
potassium voltage-gated channel subfamily A member 5 (Kv1.5) Kcna5
potassium voltage-gated channel subfamily Q member 2 Kcnq2
potassium sodium-activated channel subfamily T member 1 Kcnt1
potassium calcium-activated channel subfamily N member 2 Kcnn2
potassium calcium-activated channel subfamily M alpha 1 Kcnma1
potassium voltage-gated channel subfamily Q member 3 Kcnq3
potassium calcium-activated channel subfamily N member 3 Kcnn3
potassium calcium-activated channel subfamily N member 1 Kcnn1
potassium voltage-gated channel subfamily Q member 4 Kcnq4
potassium sodium-activated channel subfamily T member 2 Kcnt2
potassium voltage-gated channel subfamily Q member 5 Kcnq5
potassium calcium-activated channel subfamily U member 1 Kcnu1
potassium voltage-gated channel subfamily Q member 1 Kcnq1
hyperpolarization activated cyclic nucleotide gated potassium channel 3 Hcn3
hyperpolarization activated cyclic nucleotide gated potassium channel 2 Hcn2
hyperpolarization activated cyclic nucleotide gated potassium channel 1 Hcn1
hyperpolarization activated cyclic nucleotide gated potassium channel 4 Hcn4

Figure S6

Figure S7

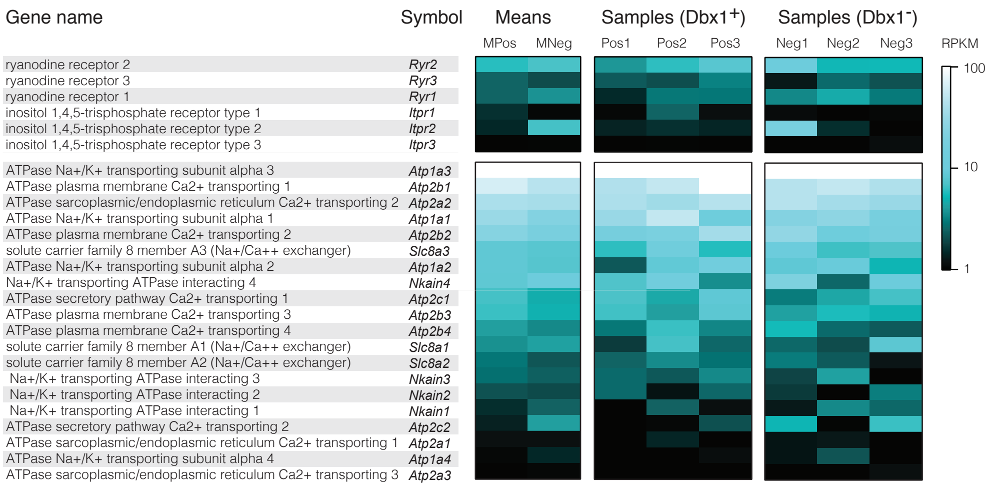

Gene name
Symbol
Means
Samples (Dbx1+)
Samples (Dbx1-)
MPos
MNeg
Pos1
Pos2
Pos3
Neg1
Neg2
Neg3
RPKM
100
10
1
ryanodine receptor 2 Ryr2
ryanodine receptor 3 Ryr3
ryanodine receptor 1 Ryr1
inositol 1,4,5-trisphosphate receptor type 1 Itpr1
inositol 1,4,5-trisphosphate receptor type 2 Itpr2
inositol 1,4,5-trisphosphate receptor type 3 Itpr3
ATPase Na+/K+ transporting subunit alpha 3 Atp1a3
ATPase plasma membrane Ca2+ transporting 1 Atp2b1
ATPase sarcoplasmic/endoplasmic reticulum Ca2+ transporting 2 Atp2a2
ATPase Na+/K+ transporting subunit alpha 1 Atp1a1
ATPase plasma membrane Ca2+ transporting 2 Atp2b2
solute carrier family 8 member A3 (Na+/Ca++ exchanger) Slc8a3
ATPase Na+/K+ transporting subunit alpha 2 Atp1a2
Na+/K+ transporting ATPase interacting 4 Nkain4
ATPase secretory pathway Ca2+ transporting 1 Atp2c1
ATPase plasma membrane Ca2+ transporting 3 Atp2b3
ATPase plasma membrane Ca2+ transporting 4 Atp2b4
solute carrier family 8 member A1 (Na+/Ca++ exchanger) Slc8a1
solute carrier family 8 member A2 (Na+/Ca++ exchanger) Slc8a2
Na+/K+ transporting ATPase interacting 3 Nkain3
Na+/K+ transporting ATPase interacting 2 Nkain2
Na+/K+ transporting ATPase interacting 1 Nkain1
ATPase secretory pathway Ca2+ transporting 2 Atp2c2
ATPase sarcoplasmic/endoplasmic reticulum Ca2+ transporting 1 Atp2a1
ATPase Na+/K+ transporting subunit alpha 4 Atp1a4
ATPase sarcoplasmic/endoplasmic reticulum Ca2+ transporting 3 Atp2a3

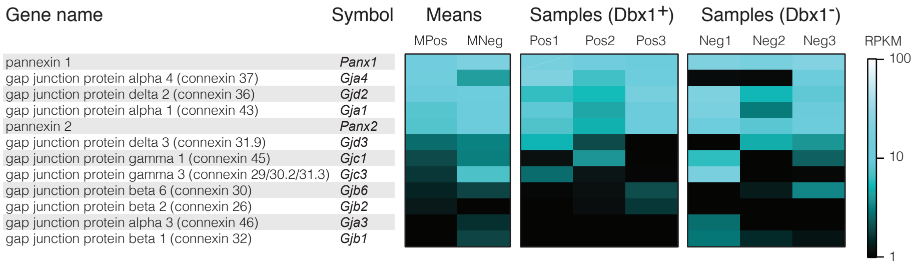
Gene name
Symbol
Means
Samples (Dbx1+)
Samples (Dbx1-)
MPos
MNeg
Pos1
Pos2
Pos3
Neg1
Neg2
Neg3
RPKM
100
10
1
pannexin 1
Panx1
gap junction protein alpha 4 (connexin 37)
Gja4
gap junction protein delta 2 (connexin 36)
Gjd2
gap junction protein alpha 1 (connexin 43)
Gja1
pannexin 2
Panx2
gap junction protein delta 3 (connexin 31.9)
Gjd3
gap junction protein gamma 1 (connexin 45)
Gjc1
gap junction protein gamma 3 (connexin 29/30.2/31.3)
Gjc3
gap junction protein beta 6 (connexin 30)
Gjb6
gap junction protein beta 2 (connexin 26)
Gjb2
gap junction protein alpha 3 (connexin 46)
Gja3
gap junction protein beta 1 (connexin 32)
Gjb1

Figure S8

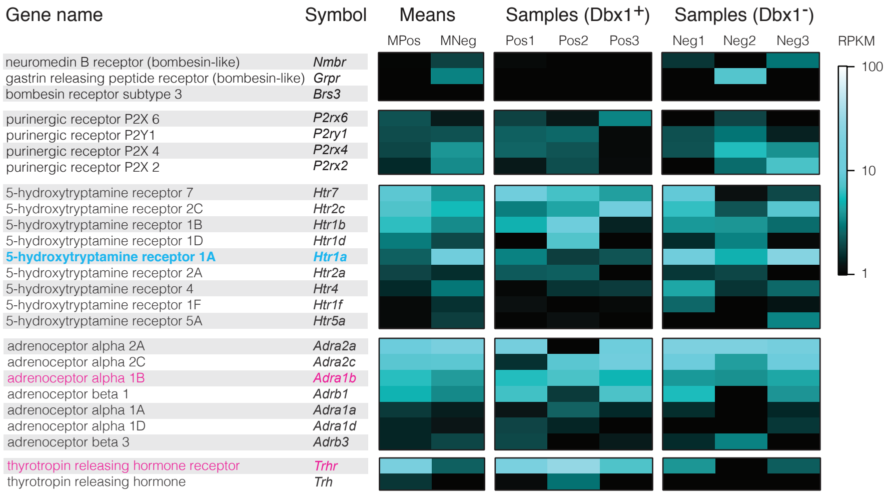
Gene name
Symbol
Means
Samples (Dbx1+)
Samples (Dbx1-)
MPos
MNeg
Pos1
Pos2
Pos3
Neg1
Neg2
Neg3
RPKM
100
10
1
neuromedin B receptor (bombesin-like) Nmbr
gastrin releasing peptide receptor (bombesin-like) Grpr
bombesin receptor subtype 3 Brs3
purinergic receptor P2X 6 P2rx6
purinergic receptor P2Y1 P2ry1
purinergic receptor P2X 4 P2rx4
purinergic receptor P2X 2 P2rx2
5-hydroxytryptamine receptor 7 Htr7
5-hydroxytryptamine receptor 2C Htr2c
5-hydroxytryptamine receptor 1B Htr1b
5-hydroxytryptamine receptor 1D Htr1d
5-hydroxytryptamine receptor 1A Htr1a
5-hydroxytryptamine receptor 2A Htr2a
5-hydroxytryptamine receptor 4 Htr4
5-hydroxytryptamine receptor 1F Htr1f
5-hydroxytryptamine receptor 5A Htr5a
adrenoceptor alpha 2A Adra2a
adrenoceptor alpha 2C Adra2c
adrenoceptor alpha 1B Adra1b
adrenoceptor beta 1 Adrb1
adrenoceptor alpha 1A Adra1a
adrenoceptor alpha 1D Adra1d
adrenoceptor beta 3 Adrb3
thyrotropin releasing hormone receptor Trhr
thyrotropin releasing hormone Trh

Figure S9
